# Programme choice for perimetry in neurological conditions (PoPiN): a systematic review of perimetry options and patterns of visual field loss

**DOI:** 10.1186/s12886-018-0912-1

**Published:** 2018-09-10

**Authors:** Lauren R. Hepworth, Fiona J. Rowe

**Affiliations:** Department of Health Services Research, University of Liverpool, Waterhouse Building, Block B, First Floor1-5 Brownlow Street, Liverpool, L69 3GL UK

**Keywords:** Perimetry, Visual field loss, Idiopathic intracranial hypertension, Chiasmal compression, Stroke, Optic neuropathy

## Abstract

**Background:**

Visual field loss occurs frequently in neurological conditions and perimetry is commonly requested for patients with suspected or known conditions. There are currently no guidelines for how visual fields in neurological conditions should be assessed. There is a wide range of visual field programs available and the wrong choice of program can potentially fail to detect visual field loss. We report the results of a systematic review of the existing evidence base for the patterns of visual field loss in four common neurological conditions and the perimetry programs used, to aid the design of future research and clinical practice guidelines.

**Methods:**

A systematic search of the literature was performed. The inclusion criteria required studies testing and/or reporting visual field loss in one or more of the target conditions; idiopathic intracranial hypertension, optic neuropathy, chiasmal compression and stroke. Scholarly online databases and registers were searched. In addition articles were hand searched. MESH terms and alternatives in relation to the four target conditions and visual fields were used. Study selection was performed by two authors independently. Data was extracted by one author and verified by a second.

**Results:**

This review included 330 studies; 51 in relation to idiopathic intracranial hypertension, 144 in relation to optic neuropathy, 105 in relation to chiasmal compression, 21 in relation to stroke and 10 in relation to a mixed neuro-ophthalmology population.

**Conclusions:**

Both the 30–2 and 24–2 program using the Humphrey perimeter were most commonly reported followed by manual kinetic perimetry using the Goldmann perimeter across all four conditions included in this review. A wide variety of other perimeters and programs were reported. The patterns of visual field defects differ much more greatly across the four conditions. Central perimetry is used extensively in neurological conditions but with little supporting evidence for its diagnostic accuracy in these, especially considering the peripheral visual field may be affected first whilst the central visual field may not be impacted until later in the progression. Further research is required to reach a consensus on how best to standardise perimetry for neurological conditions.

**Electronic supplementary material:**

The online version of this article (10.1186/s12886-018-0912-1) contains supplementary material, which is available to authorized users.

## Background

Perimetry is the systematic measurement of visual field function using different types and intensities of stimuli. Visual fields may be assessed by using moving (kinetic) targets which outline the boundaries of visual field or by using static (stationary on-off) targets which map the sensitivity within the visual field [1]. The visual field is the full area which can be seen by each eye and includes both central and peripheral vision.

Perimetry programs can be chosen to measure the central or peripheral visual field, or both [1]. Typically, the central visual field is assessed as approximately 60% of all retinal nerve fibres originate from the central 30 degrees of the visual field [1]. Therefore, assessment of the central visual field tends to show the majority of visual field loss caused by common ophthalmic disease/conditions. Peripheral visual field assessment is indicated where pathology is known to affect the visual field outside the central 30 degrees.

Visual field assessment is an important clinical tool in the assessment of patients with acute and chronic ocular and/or neurological diseases and is often considered a ‘corner-stone’ assessment in ophthalmology services. Glaucoma is the most common ocular condition for which visual field assessment is required [2]. Visual field assessment using standard automated perimetry with a central thresholding test is listed as a key priority for implementation in the diagnosis of glaucoma [3]. Specifically the 24–2 program is referred to as the reference standard in assessing visual fields [3].

Given the choice of many perimetry programs across a variety of perimeters on the market, it is important to understand the designs of the programs available and apply them according to the type of visual field loss expected in order to improve diagnostic accuracy. In neuro-ophthalmology, perimetry has three important functions: 1) diagnostic, 2) monitoring and 3) functional assessment [4].

Diagnostic accuracy is important for any condition affecting the visual pathway particularly as a missed diagnosis of visual field loss can delay diagnosis of neurological pathology with serious life consequences. The recommendation for the 24–2 programme in glaucoma has streamlined clinical practice, allowing interchange of results across hospitals and providing a clinical result that clinicians worldwide recognise and accept. Such significant practice must be applied to other commonly occurring conditions to afford the same benefits.

It is not yet known how best to assess the visual field of individuals with neurological conditions. As visual field loss occurs frequently in neurological conditions, perimetry is commonly requested at eye clinics for patients with suspected or known diagnoses. There are currently no guidelines for how visual fields in neurological conditions should be assessed. There is a pressing need to identify reference standard visual field program for neurological conditions.

The aim of this study is to undertake a systematic review of the existing evidence base for perimetry in common neurological conditions. This will aid the design of future research and clinical practice guidelines. The primary objective is to determine the common patterns of visual field defects in chiasmal compression, idiopathic intracranial hypertension (IIH), stroke and optic neuropathy, and the secondary objective is to identify the common perimeters and visual field programmes used to investigate these conditions.

## Methods

This review was registered with PROSPERO [Ref: CRD42017080742] [5].

### Types of studies

The following types of studies were included in the review: randomised controlled trials, controlled trials, prospective and retrospective cohort studies, observational studies and case controlled studies. Case reports, editorials and letters were excluded. All languages were included and translations were obtained when necessary. Studies of participants reporting visual field loss relating to chiasmal compression, IIH, stroke and optic neuropathy were included. The search was limited to publications after 1990; this date restriction was chosen to coincide with the switch to the use of the Humphrey Field Analyser II-*i* Series which is still currently and commonly used within ophthalmology clinics.

### Target conditions

Common neurological conditions of IIH, optic neuropathies, chiasmal compression and stroke were targeted [2].

In IIH, loss of visual function may occur at any stage [6]. Monitoring of visual fields is crucial in this population as visual loss can be insidious and asymptomatic for a considerable amount of time [7]. The frequency of subclinical visual loss underscores the need for thorough ophthalmological examination with perimetry [8].

Two common optic neuropathies include optic neuritis and anterior ischaemic optic neuropathy (AION), however there are many other optic neuropathy aetiologies [7]. Visual field loss in optic neuropathy is an important factor in diagnosis [9]. Within this review the following types of optic neuropathy were included: AION, non-arteritic anterior ischaemic optic neuropathy (NAION), optic neuritis, thyroid/Grave’s, toxic and traumatic.

Visual field loss is a common mode of presentation for chiasmal compression. There is clinical significance to the detection of visual field loss in chiasmal compression and capturing peripheral loss is important to early diagnosis, which is essential to allow prompt neurosurgical intervention [10].

The prevalence of visual field loss following stroke has been reported in approximately one third of stroke survivors [11]. UK national guidelines recommend that every patient with stroke be examined for the presence of visual field loss [12]. Repeated perimetry in stroke-related visual field loss is important to track recovery [13].

### Information sources and search strategy

A systematic strategy to search key electronic databases, including Cochrane registers and electronic bibliographic databases was used: Cochrane Stroke Group Trials Register, Cochrane Eyes and Vision Group Trials Register, Cochrane Central Register of Controlled Trials (CENTRAL), MEDLINE, EMBASE, CINAHL, AMED, PsycINFO, Dissertations & Theses (PQDT) database, British Nursing Index, PsycBITE (Psychological Database for Brain Impairment Treatment Efficacy), ClinicalTrials.gov, Current Controlled Trials, Trials Central, Health Service Research Projects in Progress, National Eye Institute Clinical Studies Database, Orthoptic Search Facility and Proceedings of Association for Research in Vision and Ophthalmology. Search terms are detailed in Table 1.

**Table 1:** Search terms

OR	OR
AND	
Pituitary	Visual Fields
Pituitary adenoma	Vision Disorders
Craniopharyngioma	Vision
Pseudotumour cerebri	Visual field loss
Idiopathic intracranial hypertension	Visual field defect
Benign intracranial hypertension	Perimetry
Chiasm	Perimeter
Stroke	Visual field assessment
Cerebrovascular disorders	Humphrey™
Brain ischaemia	Octopus™
Intracranial Haemorrhage	
Optic neuropathy	
Anterior ischaemic optic neuropathy	
Multiple sclerosis	
Optic neuritis	
Demyelination	
Neuromyelitis optica	
Devic’s disease	
Compressive neuropathy	
Toxic neuropathy	

™ Humphrey (Carl Zeiss AG, Germany), Octopus (Haag Streit International, Switzerland)

### Selection process and quality assessment

The titles and abstracts identified from the search were independently screened by the two authors through each phase of the review (screening, eligibility and inclusion) using the pre-stated inclusion criteria. The full papers of any studies considered potentially relevant were considered and the selection criteria applied independently by two reviewers. We resolved disagreements at each step by discussion between the two review authors; all were solved in this manner without the need to seek the opinion of a third reviewer.

The data being extracted from the studies was not related to the study methodology, therefore quality assessment of the individual studies was not required.

### Data extraction for included studies

A pre-designed data extraction form was used to gather information on sample size, study design, defect type, severity and location and choice of visual field program. The data was extracted and documented by one researcher (LH) and verified by another (FR).

## Results

**Fig. 1:**
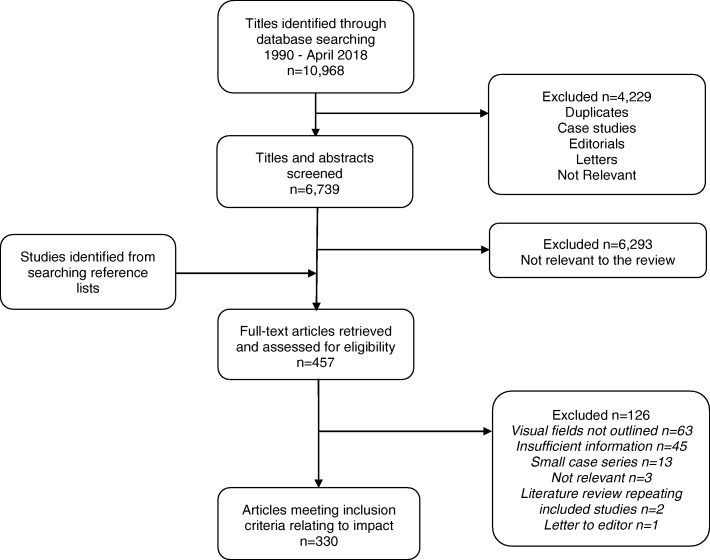
Flowchart of pathway for inclusion of articles

The search results are outlined in Fig. 1. Three hundred and thirty studies were included. Fifty-one of the studies reported on IIH, 144 studies reported on optic neuropathy, 105 studies reported on chiasmal compression, 21 studies reported on stroke and 10 studies reported on a mixed neuro-ophthalmology population.

The most commonly used perimeters and programmes for IIH, optic neuropathy, chiasmal compression and stroke are outlined in Table 2.

**Table 2:** Number of most commonly reported perimeters and programmes used for the four neurological conditions of interest, including if more detail on programme was specified or not. ™ Humphrey (Zeiss Meditec, USA), Octopus (Haag Streit International, Switzerland)

n=		Neurological condition			
		Idiopathic intracranial hypertension	Optic Neuropathy	Chiasmal compression	Stroke
Perimeter used	Multiple	26	27	31	5
	Humphrey	38	86	60	11
		10 unspecified 28 specified	5 unspecified 81 specified	7 unspecified 51 specified	3 unspecified 8 specified
	Humphrey 30-2	16	46	20	4
		6 unspecified 10 specified	22 unspecified 24 specified	10 unspecified 10 specified	0 unspecified 4 specified
	Humphrey 24-2	14	39	22	5
		7 unspecified 7 specified	11 unspecified 28 specified	5 unspecified 17 specified	1 unspecified 4 specified
	Goldmann	22	31	41	8
		18 unspecified 4 specified	30 unspecified 1 specified	41 unspecified	7 unspecified 1 specified
	Octopus	6	9	9	1
		6 unspecified	1 unspecified 8 specified	2 unspecified 7 specified	1 unspecified

**Fig. 2:**
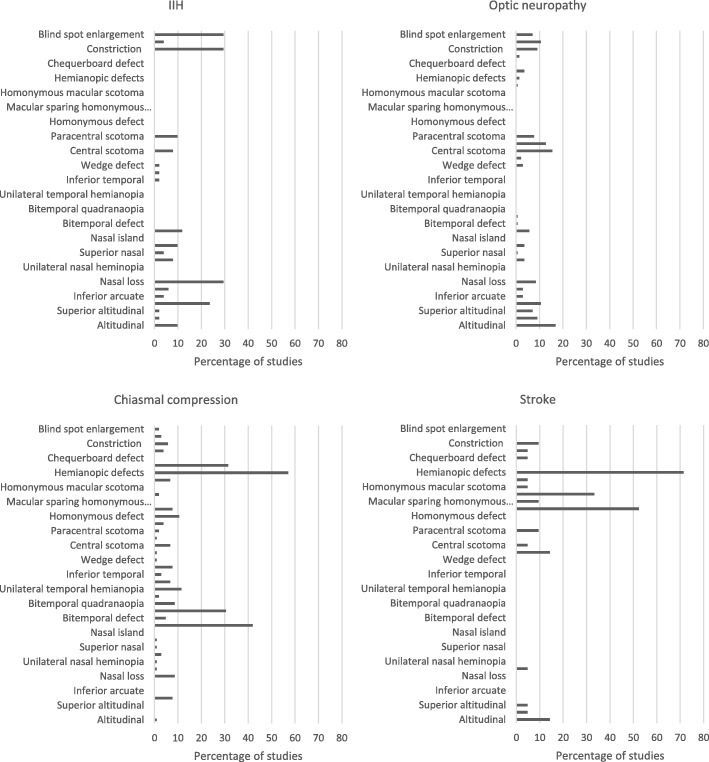
Percentage of reported patterns of visual field loss in the four neurological conditions of interest

All the reported patterns of visual field loss for IIH, optic neuropathy, chiasmal compression and stroke are outlined in Fig. 2.

For the purposes of identifying perimetry programs, papers which were clearly associated with the same study i.e. Idiopathic Intracranial Hypertension Treatment Trial (IIHTT)^[23–27]^ and Optic Neuritis Treatment Trial (ONTT)^[19, 28–40]^, the study was counted once as the same protocol applied to all papers.

### Idiopathic intracranial hypertension

#### Perimetry choices

Of the 44 studies reporting visual field testing in IIH, the majority (*n* = 38) of studies reported using a Humphrey perimeter^[8, 23, 41–76]^. Of the studies which reported the specific program there was an almost even split between the 30–2 (*n* = 16)^[41–43, 47, 52, 60–62, 64–66, 71–73, 75, 76]^ and 24–2 (*n* = 14)^[8, 23, 41, 43–46, 56, 57, 64, 67–69, 73]^ programs. The stategy used in these two programs was a mixture of full threshold^[46]^, SITA Standard^[23, 24, 52, 65–69, 72, 75]^ and SITA Fast^[49, 60–62, 70]^. The use of the Goldmann perimeter was reported by 22 studies^[8, 43, 45, 46, 48, 50, 51, 53–55, 58, 59, 62, 63, 67, 72, 76–81]^ and the Octopus perimeter in six studies^[59, 70, 76, 78–80]^. The variety of specific programs used on the Octopus perimeter included 32 program^[78]^, 24 program^[78]^, 30 degree static^[76]^, 90 degree static^[79]^ and kinetic^[70]^. Twenty-six studies reported the use of multiple perimeters and/or programs^[8, 41, 43–46, 48–51, 53–55, 58, 59, 62–64, 67, 70, 72, 73, 76, 78–80, 82]^, with two citing an indication such as poor vision or concentration^[62, 72]^ for using an alternative and a further two changing perimeter or program at a set time point^[76, 80]^.

A variety of other perimeters/perimetry included motion periemtry^[41, 44]^, Tangent screen^[43, 49]^, high pass resolution (Ophthimus)^[44]^, Rarebit perimetry^[82]^ and macro automated MP 30–2 ^[82]^.

#### Patterns of visual field loss

The most common patterns of visual field loss reported by the included studies were blind spot enlargement (*n* = 15)^[41, 42, 47, 48, 53, 54, 58, 64, 77–81, 83, 84]^, constriction (*n* = 15)^[8, 24, 42, 47–49, 53, 54, 58, 64, 76, 77, 80, 83, 84]^, nasal loss (*n* = 15)^[8, 41, 42, 47–49, 53, 54, 58, 64, 76, 81, 83–85]^ and arcuate defects (*n* = 12)^[8, 24, 41, 48, 53, 54, 58, 64, 76, 80, 84, 85]^. Other patterns of loss reported included altitudinal (*n* = 5)^[41, 42, 53, 54, 64]^, nasal step (*n* = 5)^[8, 48, 49, 64, 85]^, paracentral scotoma (*n* = 5)^[8, 48, 54, 64, 74]^, temporal loss (*n* = 5)^[42, 53, 64, 81, 83]^ and central scotoma (*n* = 4)^[41, 54, 64, 74]^.

### Optic neuropathy

#### Perimetry choices

Of the 129 studies reporting visual field testing in optic neuropathy, the majority (*n* = 86) of studies used a Humphrey perimeter^[32, 86–170]^. Of the studies which reported the specific program the majority used the 30–2 program (*n* = 46)^[32, 86–88, 94–97, 106–120, 127–149]^ compared to the 24–2 program (*n* = 39)^[89–91, 98–102, 111, 117, 119, 121–126, 135, 139, 150–168, 170]^. The stategy used in these two programs was a mixture of full threshold^[39, 88, 97, 100, 101, 113–115, 121, 136]^, STATPAC^[116]^, SITA Standard^[100, 119, 120, 125, 126, 141–147, 154, 157–168]^, SITA Fast^[148, 149, 169]^ and short wavelength automated perimetry (SWAP)^[97, 128]^. The use of the Goldmann perimeter was reported by 31 studies^[9, 32, 92, 93, 98, 100, 103–105, 108, 130, 171–190]^. The Octopus perimeter was reported in nine studies^[178, 179, 191–197]^, and a variety of specific programs were reported; 32 program^[179]^, 24 program^[192]^, 07 program^[191]^, G program^[178, 197]^, 30^○[193, 195]^, 60^○[195]^, 90^○[196]^ static and kinetic/semi-kinetic^[194]^. Twenty-eight studies reported the use of multiple perimeters and/or programs^[32, 87, 92, 93, 97, 98, 100, 103–105, 108, 111, 117, 119, 128, 130, 135, 139, 175, 177–180, 185, 194, 195, 198]^; two cited indications of poor vision^[194]^ or concentration^[178]^ for using an alternative.

A variety of other perimeters/perimetry were reported; motion perimetry, Tangent screen^[179]^, high pass resolution (Ophthimus) ^[199, 200]^, 4–28^○^ program ^[201]^, frequency doubling perimetry^[178]^ including the C-20^[87]^ and N-30^[97]^ programs, Bjerrum screen^[202, 203]^, Tuebingen perimeter 30^○^ static^[194, 204]^ and manual^[194]^, Metrovision 30^○^ static^[205]^ and Amsler grid^[175, 180, 185]^.

#### Patterns of visual field loss

The most common patterns of visual field loss reported by the included studies were altitudinal (*n* = 24)^[9, 28, 98, 99, 103, 109, 122, 124, 126, 154, 155, 160, 168, 171, 174, 178, 180, 186, 187, 194, 206–209]^, central scotoma (*n* = 22)^[9, 28, 93, 99, 103, 118, 127, 168, 171, 174, 178, 180, 186, 187, 190, 194, 203, 206, 208–211]^, cecocentral scotoma (*n* = 18)^[9, 28, 93, 109, 116, 118, 122, 127, 155, 168, 178, 180, 194, 206, 209, 210]^, arcuate (*n* = 15)^[9, 28, 93, 98, 99, 109, 126, 154, 155, 171, 178, 181, 203, 206, 208]^ and diffuse depression (n = 15)^[28, 99, 109, 118, 122, 154–156, 160, 168, 178, 180, 194, 199, 207]^. Other patterns of loss reported included constriction (*n* = 13)^[9, 37, 93, 98, 103, 152, 155, 168, 171, 180, 186, 203, 210]^, blind spot enlargement (*n* = 10)^[9, 37, 93, 109, 171, 178, 181, 194, 208]^, nasal step (*n* = 5)^[9, 28, 93, 98, 109]^, quadrant defect (*n* = 5)^[34, 93, 152, 168, 206]^ and wedge defect (*n* = 4)^[9, 109, 194, 203]^.

### Chiasmal compression

#### Perimetry choices

Of the 105 studies reporting visual field testing in chiasmal compression, the majority (*n* = 58) of studies used a Humphrey perimeter^[10, 13, 212–267]^. Of the studies which reported the specific program the majority used the 24–2 program (*n* = 22)^[213, 214, 216, 217, 221, 224–226, 231–242, 250, 268]^ compared to the 30–2 program (*n* = 20)^[10, 212, 216, 220–223, 229, 230, 245–255]^. The strategy used in these two programs was a mixture of full threshold^[213, 223, 225, 232–234, 248–252]^, STATPAC^[214]^, FASTPAC^[226]^, SITA Standard^[232, 237, 239–242, 254, 258–267]^, SITA Fast^[255, 268]^ and SWAP^[230]^. Another program reported for the Humphrey perimeter was the 10–2 program^[224]^. The use of the Goldmann perimeter was reported by 41 studies^[214, 215, 218–220, 225, 227, 232, 244, 266, 269–299]^. The Octopus perimeter was reported in nine studies^[10, 244, 247, 269, 288, 294, 295, 300, 301]^; a variety of specific programs were reported including 32 program^[300]^, 24 program^[300]^, 30^○^ static^[295, 301]^ and kinetic/semi-kinetic^[10]^. Thirty-one studies reported the use of multiple perimeters and/or programs^[10, 214–216, 218–221, 224, 225, 227, 230, 232, 238, 244, 247, 250, 253, 266, 269–271, 273, 288, 292, 294, 295, 300, 302–304]^; four cited indications of poor vision^[304]^, concentration^[273]^, symptoms^[224]^ or diagnosis^[221]^ for using an alternative.

A variety of other perimeters/perimetry were reported; Bjerrum screen^[218]^, camprimetry^[294, 295]^, frequency doubling perimetry^[232]^, C-20^[221, 238]^ and 20–1^[238]^, high pass resolution 30^○^ (Ophthimus)^[214, 303]^, Metrovision kinetic^[305]^, Metrovision STAT 95 30^○[306]^, motion perimetry, Rarebit perimetry 24^○[303]^, Tangent screen^[271]^, Topcon perimter^[273]^, Tuebingen perimeter 30^○[307, 308]^ and Vision monitor 30^○[270]^.

#### Patterns of visual field loss

The most common patterns of visual field loss reported by the included studies were bitemporal hemianopia (*n* = 32)^[213, 217, 218, 223, 224, 226, 229, 238, 239, 243, 246, 248, 255, 258, 261, 273, 275, 276, 279, 285, 286, 289, 291, 297, 299, 304, 305, 309–313]^, other temporal loss (*n* = 21)^[215, 217, 218, 229, 238, 245, 250, 253, 255, 256, 258, 261, 269, 271, 274, 279, 294, 302, 305, 312, 313]^ and unilateral temporal hemianopia (*n* = 12)^[217, 218, 220, 246, 258, 279, 280, 289, 291, 309–311]^. Other patterns of loss reported included nasal loss (*n* = 9)^[214, 215, 217, 255, 256, 274, 279, 302, 314]^, bitemporal quadrantanopia (*n* = 9)^[229, 253, 275, 279, 285, 289, 309, 310, 313]^, arcuate (*n* = 8)^[214, 215, 217, 256, 274, 275, 311, 312]^, homonymous hemianopia (n = 8)^[218, 224, 255, 258, 279, 291, 302, 313]^, central scotoma (*n* = 7)^[218, 224, 246, 256, 274, 302, 313]^, three-quadrant loss (*n* = 7)^[223, 254, 258, 273, 285, 293, 311]^, unilateral temporal quadrantanopia (*n* = 7)^[217, 258, 289, 309–312]^ and constriction (*n* = 6)^[214, 215, 218, 274, 296, 302]^.

### Stroke

#### Perimetry choices

Of the 21 studies reporting visual field testing in stroke, the majority (*n* = 11) of studies reported using a Humphrey perimeter^[21, 315–325]^. Of the studies which reported the specific program there was an almost even split between the 24–2 program (*n* = 5)^[318–321, 323]^ and the 30–2 program (*n* = 4)^[21, 319, 320, 324]^. The strategy used in these two programs was a mixture of full threshold^[318–320]^, SITA Standard^[319–321]^ and SITA Fast^[319, 320, 324]^. Other programs used on the Humphrey perimeter were the 10–2^[323]^, 76 supra-threshold screening^[315]^, full field 120^[318]^ and Esterman^[318]^ programs. Ten studies reported the use of multiple perimeters and/or programs^[21, 316–319, 321–323, 325, 326]^, with two changing perimetry/program at a set time point^[319, 320]^.

The use of the Goldmann perimeter was reported by eight studies^[21, 316, 317, 319, 322, 325, 327, 328]^ and the Octopus perimeter by two studies^[325, 329]^.

A variety of other perimeters/perimetry were reported; Competer 750^[330]^, Humphrey Matrix ZEST^[321]^, ShP-31^[331]^, Peritest semi-automated^[326]^ and Tangent screen^[322, 332]^.

#### Patterns of visual field loss

The most common patterns of visual field loss reported by the included studies were homonymous hemianopia (*n* = 11)^[317–320, 322, 324–327, 333, 334]^ and homonymous quadrantanopia (*n* = 7)^[318, 322, 324–327, 334]^. Other patterns of loss were reported including altitudinal^[322, 325, 334]^, temporal crescent^[319, 325, 331]^ (*n* = 3), constriction^[325, 331]^, paracentral scotoma^[329, 331]^ (*n* = 2), binasal heminaopia^[325]^, central scotoma^[331]^, chequerboard loss^[325]^, homonymous macular scotoma^[327]^, unilateral blindness^[325]^, 3-quadrant loss^[325]^ (*n* = 1).

### Mixed neuro-ophthalmology population

#### Perimetry choices

Of the 10 studies reporting visual field testing in mixed neuro-ophthalmology populations, the majority (*n* = 9) of studies reported using a Humphrey perimeter^[4, 335–342]^. Of the studies which reported the specific program there was an even split between the 30–2 program (*n* = 5)^[335–337, 339, 340]^ and the 24–2 program (*n* = 5)^[337–339, 341, 342]^. The strategy used these two programs was a mixture of full threshold^[337, 339]^, FASTPAC^[337]^, SITA Standard^[339, 340]^, SITA Fast^[4, 339]^ and SWAP^[336]^. Other programs used on the Humphrey perimeter were the peripheral 68 and full field 120 programs^[335]^.

The use of the Goldmann perimeter was reported by five studies^[4, 335, 337–339]^, and the Octopus perimeter by three studies^[335, 337, 338]^. The variety of specific programs used on the Octopus perimeter included 32 program^[335, 337]^, 07 program^[335]^, G program^[337]^ and TOP program^[338]^.

A variety of other perimeters/perimetry were reported; frequency doubling perimetry^[4, 337]^, high pass resolution (Ophthimus)^[337]^ and Humphrey Matrix 30–2^[340]^.

The super-scripted references included in this meta-analysis are listed in full in Additional file 1.

### Discussion

Across all four neurological conditions a wide variety of perimeters and perimetry programs are being used. It is clear from these findings that there is no standardisation for assessment of visual fields for the neurological conditions (IIH, optic neuropathy, chiasmal compression and stroke) at the focus of this review.

The majority of studies reported using the Humphrey perimeter. The Humphrey II-*i* Series mainly performs static perimetry programs, both central and peripheral. Kinetic perimetry was available on the 750i model and was optional on 740i and 745i models [14]. The most commonly used static perimetry programs were the 30–2 and 24–2. Both these programs assess the central portion of the visual field. The 30–2 program assesses a grid of 76 points over the central 30° of the visual field. The 24–2 program has limitations in that its assessment of visual field is restricted on superior, inferior and temporal sides to 24° with an extension to 27° nasally, assessing a total grid of 54 points [1]. As a result, it can miss visual field loss outside these extremities leading to poor diagnostic accuracy in certain conditions [10, 15]. Although static automated perimetry has been shown to be adequate in neuro-ophthalmology practice, kinetic perimetry is useful for patients with severe visual and neurological deficits and patients with peripheral visual field defects [16, 17].

The second most commonly reported perimeter was the Goldmann perimeter, used in 100 of the included studies. The Goldmann perimeter is primarily used to perform manual kinetic perimetry [1]. In addition to the use of the Goldmann perimeter, a number of studies reported using semi-kinetic/kinetic perimetry using the Octopus 900 perimeter. The Octopus 900 perimeter was the replacement for the Goldmann perimeter when Goldmann production ceased in 2007. The Octopus 900 is capable of performing both kinetic and static perimetry programs. Kinetic perimetry programs can be pre-set and run as an automatic program or performed manually in the equivalent way as Goldmann kinetic perimetry. A comparison of kinetic perimetry using the Goldmann and Octopus perimeters, found strong agreement in detecting the presence of all visual field defects for type and location of defect between the two instruments [17].

Comparative studies have contrasted different combinations of static and kinetic perimetry in single and mixed neuro-ophthalmic conditions. Szatmáry and colleagues compared the Humphrey SITA Fast 24–2 program to Goldmann manual perimetry in a mixed neuro-ophthalmic population, with similar defects found on both tests in 61.5% [18]. The authors concluded for central defects the SITA Fast program may be useful but the development of program extending further into the peripheral visual field would be more appropriate for neuro-ophthalmology [18]. Rowe and colleagues compared a Humphrey peripheral static screening program (full field 120) to an Octopus peripheral kinetic strategy in a mixed neuro-ophthalmology population [16]. A match for normal or abnormal visual field results was reported for 87% of the cases. The authors concluded that although the full field 120 was useful for detection of visual field defects, Octopus kinetic perimetry was advantageous providing added information of defect depth and size plus a more representative view of the visual field defect [16]. Keltner and colleagues compared central (30–2 program) and peripheral (manual kinetic) perimetry in optic neuritis, they reported a greater number of visual field defects were within the central area (97.1%) compared to the peripheral area (69.9%) at baseline [19]. The authors concluded in the majority of cases optic neuritis could be monitored using a central program, but in more severe cases peripheral perimetry would be required [19]. Rowe et al. compared the Humphrey 30–2 and 24–2 programs and Octopus semi-automated kinetic perimetry in a population with pituitary disease, they reported kinetic perimetry to be the favoured option when available and recommends the 30–2 over the 24–2 program in this population [10]. Wong and colleagues compared the Goldmann perimeter (manual kinetic), Humphrey perimeter (30–2 program) and tangent screen (manual kinetic) for the detection and localisation of occipital lesions [20]. The detection of visual field defects was achieved by all three techniques, however the Humphrey 30–2 program failed to be in agreement in 33% of cases in terms of localisation. This study also reported the 10–2 program detection of macular sparing was in agreement with that of the manual kinetic perimetry. The authors concluded all were suitable for screening, however more information was provided by kinetic perimetry [20]. Pineles and colleagues compared an automated combined static and kinetic program using an Octopus perimeter to standard static (24–2 or 30–2 programs) or Goldmann manual perimetry in a mixed neuro-ophthalmic population, 86% of visual field defects matched [21]. The authors argued that the combination of both static and kinetic perimetry overcome the limitations the individual types of perimetry [21]. These comparative studies have highlighted there are advantages and disadvantages within the range of available perimetry options.

The most commonly reported patterns of visual field defect for IIH included arcuate (predominantly superiorly), constriction and blind-spot enlargement.The patterns of visual field defect most commonly reported in cases of optic neuropathy are the most diverse of the four conditions; these were altitudinal defects, central, cecocentral and paracentral scotomas, diffuse depression, arcuate defects and constriction. The most commonly reported patterns of visual field defect for chiasmal compression included hemianopic and quadrantanopic defects, predominantly to the temporal side. In the case of stroke, the most commonly reported patterns of visual field defect were homonymous hemianopic and quadrantanopic defects.

With the expection of optic neuropathy and IIH, the majority of patterns of visual field defects reported are peripheral defects. The 30–2 program and equivalents detect the presence of central defects however, do not show peripheral defects so cannot display the full extent of the visual field loss and may not detect visual field loss until it is further advanced such that it also affects the central field.

A limitation of this review was the restriction of targeting four common neurological conditions which cause visual field loss. Furthermore the types of optic neuropathy were also limited. It is therefore not inclusive of all neurological conditions.

## Conclusion

The common perimeter programs and the common patterns of visual field defect and for IIH, optic neuropathy, chiasmal compression and stroke have been reported. Both the 30–2 and 24–2 program using the Humphrey perimeter are most commonly reported followed by manual kinetic perimetry using the Goldmann perimeter across all four conditions included in this review. The patterns of visual field defects reported differ much more greatly across the four conditions. In IIH, blind spot enlargement, constriction, nasal loss and arcuate defects were most commonly reported. In optic neuropathy, altitudinal defects, arcuate defects, diffuse depression, central and cecocentral scotomas were most commonly reported. In chiasmal compression, the most commonly reported were bitemporal hemianopia, unilateral temporal hemiaopia and other temporal defects. In stroke, homonymous hemianopia and quadrantaopia were the most commonly reported defects.

It is apparent that the 24–2 perimetry strategy is used extensively for visual field assessment in neurological conditions but with little supporting evidence for its diagnostic accuracy in these particularly where visual field loss may affect the peripheral visual field first and may not impact the central visual field until later in the progression, if at all. It is important now to research this topic further in order to reach consensus on how best to standardise perimetry for neurological conditions.

## Additional file

Additional file 1: Meta-analysis references. (DOC 131 kb)

## Abbreviations

AION: Anterior ischaemic optic neuropathy
IIH: Idiopathic intercranial hypertension
NAION: Non-arteritic anterior ischaemic optic neuropathy
