# Supplementary material for: Programme choice for perimetry in neurological conditions (PoPiN): a systematic review of perimetry options and patterns of visual field loss
Source: BMC Ophthalmol. 2018 Sep 10;18:241. doi: 10.1186/s12886-018-0912-1 (PMC6131852; doi:10.1186/s12886-018-0912-1)
Supplement: Supplementary file 1 — Meta-analysis references. (DOC 131 kb) [file 12886_2018_912_MOESM1_ESM.doc]

**Additional File 1:** References included in the meta-analysis, included in the manuscript as super-script.

4. Kedar S, Ghate D, Corbett JJ: Visual fields in neuro-ophthalmology. *Indian Journal of Ophthalmology* 2011, 59(2):103-9

8. Rowe FJ, Sarkies NJ: Assessment of visual function in idiopathic intracranial hypertension: a prospective study. *Eye* 1998, 12:111-8

9. Hayreh SS, Zimmerman B: Visual field abnormalities in nonarteritic anterior ischemic optic neuropathy their pattern and prevalence at initial examination. *Archives of Ophthalmology* 2005, 123:1554-62

10. Rowe FJ, Chenye CP, Garcia-Fiñana M, Noonan C, Howard C, Smith J, Adeoye J: Detection of visual field loss in pituatary disease: peripheral kinetic versus central static. *Neuro-Ophthalmology* 2015, 39(3):116-24

13. Jones SA, Shinton RA: Improving outcome in stroke patients with visual problems. *Age and Ageing* 2006, 35(6):560-5

19. Keltner JL, Johnson CA, Spurr JO, Beck RW: Comparison of central and peripheral visual field properties in the Optic Neuritis Treatment Trial. *American Journal of Ophthalmology* 1999, 128(5):543-53

21. Wong AM, Sharpe JA: A comparison of tangent screen, goldmann, and humphrey perimetry in the detection and localization of occipital lesions. *Ophthalmology* 2000, 107(3):527-44

23. Friedman DI, McDermott MP, Kieburtz K, Kupersmith M, Stoutenburg A, Keltner JL, Feldon SE, Schron E, Corbett JJ, Wall M *et al*: The Idiopathic Intracranial Hypertension Treatment Trial: design considerations and methods. *Journal of Neuro-Ophthalmology* 2014, 34(2):107-17

24. Keltner JL, Johnson CA, Cello KE, Wall M, NORDIC Idiopathic Intracranial Hypertension Study Group: Baseline visual field findings in the Idiopathic Intracranial Hypertension Treatment Trial (IIHTT). *Invest Ophthalmol Vis Sci* 2014, 55(5):3200-7

25. Wall M, Kupersmith MJ, Kieburtz KD, Corbett JJ, Feldon SE, Friedman DI, Katz DM, Keltner JL, Schron EB, McDermott MP *et al*: The Idiopathic Intracranial Hypertension Treatment Trial: Clinical profile at baseline. *JAMA Neurology* 2014, 71(6):693-701

26. Wall M, McDermott MP, Kieburtz KD, Corbett JJ, Feldon SE, Friedman DI, Katz DM, Keltner JL, Schron EB, Kupersmith MJ *et al*: Effect of acetazolamide on visual function in patients with idiopathic intracranial hypertension and mild visual loss: the Idiopathic Intracranial Hypertension Treatment Trial. *Journal of the American Medical Association* 2014, 311(16):1641-51

27. Wall M, Johnson CA, Cello KE, Zamba KD, McDermott MP, Keltner JL, Nordic Idiopathic Intracranial Hypertension Study Group: Visual field outcomes for the Idiopathic Intracranial Hypertension Treatment Trial (IIHTT). *Invest Ophthalmol Vis Sci* 2016, 57(3):805-12

28. Keltner JL, Johnson CA, Spurr JO, Beck RW: Baseline visual field profile of optic neuritis. The experience of the optic neuritis treatment trial. Optic Neuritis Study Group. *Archives of Ophthalmology* 1993, 111(2):231-4

29. Keltner JL, Johnson CA, Spurr JO, Beck RW: Visual field profile of optic neuritis. One-year follow-up in the Optic Neuritis Treatment Trial. *Archives of Ophthalmology* 1994, 112(7):946-53

30. Beck RW, Cleary PA, Optic Neuritis Study Group: Optic neuritis treatment trial: one-year follow-up results. *Archives of Ophthalmology* 1993, 111(6):773-5

31. Beck RW, Cleary PA, Backlund JC, Optic Neuritis Study Group: The course of visual recovery after optic neuritis: experience of the Optic Neuritis Treatment Trial. *Ophthalmology* 1994, 101(11):1771-8

32. Cleary PA, Beck RW, Anderson MM, Jr., Kenny DJ, Backlund JY, Gilbert PR, Optic Neuritis Study Group: Design, methods, and conduct of the Optic Neuritis Treatment Trial. *Controlled Clinical Trials* 1993, 14(2):123-42

33. Cleary PA, Beck RW, Bourque LB, Backlund JC, Miskala PH, Optic Neuritis Study Group: Visual symptoms after optic neuritis: results from the Optic Neuritis Treatment Trial. *Journal of Neuro-Ophthalmology* 1997, 17(1):18-23

34. Fang JP, Donahue SP, Lin RH: Global visual field involvement in acute unilateral optic neuritis. *American Journal of Ophthalmology* 1999, 128(5):554-65

35. Fang JP, Lin RH, Donahue SP: Recovery of visual field function in the optic neuritis treatment trial. *American Journal of Ophthalmology* 1999, 128(5):566-72

36. Beck RW, Gal RL, Bhatti MT, Brodsky MC, Buckley EG, Chrousos GA, Corbett J, Eggenberger E, Goodwin JA, Katz B *et al*: Visual function more than 10 years after optic neuritis: experience of the Optic Neuritis Treatment Trial. *American Journal of Ophthalmology* 2004, 137(1):77-83

37. Keltner JL, Johnson CA, Cello KE, Dontchev M, Gal RL, Beck RW, Optic Neuritis Study Group: Visual field profile of optic neuritis: a final follow-up report from the optic neuritis treatment trial from baseline through 15 years. *Archives of Ophthalmology* 2010, 128(3):330-7

38. Kupersmith MJ, Gal RL, Beck RW, Xing D, Miller N: Visual function at baseline and 1 month in acute optic neuritis: predictors of visual outcome. *Neurology* 2007, 69(6):508-14

39. Optic Neuritis Study Group: Visual function 15 years after optic neuritis: a final follow-up report from the Optic Neuritis Treatment Trial. *Ophthalmology* 2008, 115(6):1079-82

40. Optic Neuritis Study Group: Visual function 5 years after optic neuritis: experience of the Optic Neuritis Treatment Trial. . *Archives of Ophthalmology* 1997, 115(12):1545-52

41. Wall M, Montgomery EB: Using motion perimetry to detect visual field defects in patients with idiopathic intracranial hypertension: a comparison with conventional automated perimetry. *Neurology* 1995, 45(6):1169-75

42. Salgarello T, Tamburrelli C, Falsini B, Giudiceandrea A, Colotto A: Optic nerve diameters and perimetric thresholds in idiopathic intracranial hypertension. *British Journal of Ophthalmology* 1996, 80(6):509-14

43. Kupersmith MJ, Gamell L, Turbin R, Peck V, Spiegel P, Wall M: Effects of weight loss on the course of idiopathic intracranial hypertension in women. *Neurology* 1998, 50(4):1094-8

44. Wall M, White WN, 2nd: Asymmetric papilledema in idiopathic intracranial hypertension: prospective interocular comparison of sensory visual function. *Invest Ophthalmol Vis Sci* 1998, 39(1):134-42

45. Rowe FJ: The symptoms of raised intracranial pressure in idiopathic intracranial hypertension. *British Orthoptic Journal* 2000, 57:15-8

46. Rowe FJ, Sarkies NJ: The relationship between obesity and idiopathic intracranial hypertension. *International Journal of Obesity* 1999, 23(1):54-9

47. Craig JJ, Mulholland DA, Gibson JM: Idiopathic intracranial hypertension; incidence, presenting features and outcome in Northern Ireland (1991-1995). *Ulster Med J* 2001, 70(1):31-5

48. Rowe FJ, Noonan CP: The presenting profile of paediatric idiopathic intracranial hypertension. In: *European Strabismological Association.* Florence, Italy; 2001: 55-8.

49. Stiebel-Kalish H, Lusky M, Yassur Y, Kalish Y, Shuper A, Erlich R, Lubman S, Snir M: Swedish Interactive Thresholding Algorithm Fast for following visual fields in prepubertal idiopathic intracranial hypertension. *Ophthalmology* 2004, 111(9):1673-5

50. Bruce BB, Preechawat P, Newman NJ, Lynn MJ, Biousse V: Racial differences in idiopathic intracranial hypertension. *Neurology* 2008, 70(11):861-7

51. Bruce BB, Kedar S, Van Stavern GP, Monaghan D, Acierno MD, Braswell RA, Preechawat P, Corbett JJ, Newman NJ, Biousse V: Idiopathic intracranial hypertension in men. *Neurology* 2009, 72(4):304-9

52. Nithyanandam S, Manayath GJ, Battu RR: Optic nerve sheath decompression for visual loss in intracranial hypertension: report from a tertiary care center in South India. *Indian Journal of Ophthalmology* 2008, 56(2):115-20

53. Digre KB, Nakamoto BK, Warner JE, Langeberg WJ, Baggaley SK, Katz BJ: A comparison of idiopathic intracranial hypertension with and without papilledema. *Headache* 2009, 49(2):185-93

54. Baldwin MK, Lobb B, Tanne E, Egan R: Weight and visual field deficits in women with idiopathic intracranial hypertension. *Journal of Women's Health* 2010, 19(10):1893-8

55. Bruce BB, Kedar S, Van Stavern GP, Corbett JJ, Newman NJ, Biousse V: Atypical idiopathic intracranial hypertension: normal BMI and older patients. *Neurology* 2010, 74(22):1827-32

56. Rebolleda G, Munoz-Negrete FJ: Follow-up of mild papilledema in idiopathic intracranial hypertension with optical coherence tomography. *Invest Ophthalmol Vis Sci* 2009, 50(11):5197-200

57. Sinclair AJ, Burdon MA, Nightingale PG, Ball AK, Good P, Matthews TD, Jacks A, Lawden M, Clarke CE, Stewart PM *et al*: Low energy diet and intracranial pressure in women with idiopathic intracranial hypertension: prospective cohort study. *British Medical Journal* 2010, 341(c2701) DOI: 10.1136/bmj.c2701.

58. Baheti NN, Nair M, Thomas SV: Long-term visual outcome in idiopathic intracranial hypertension. *Annals of Indian Academy of Neurology* 2011, 14(1)

59. Rowe FJ: Assessment of visual function in idiopathic intracranial hypertension. *Br J Neurosurg* 2011, 25(1):45-54

60. Skau M, Yri H, Sander B, Gerds TA, Milea D, Jensen R: Diagnostic value of optical coherence tomography for intracranial pressure in idiopathic intracranial hypertension. *Graefes Arch Clin Exp Ophthalmol* 2013, 251(2):567-74

61. Skau M, Sander B, Milea D, Jensen R: Disease activity in idiopathic intracranial hypertension: a 3-month follow-up study. *Journal of Neurology* 2011, 258(2):277-83

62. Soiberman U, Stolovitch C, Balcer LJ, Regenbogen M, Constantini S, Kesler A: Idiopathic intracranial hypertension in children: visual outcome and risk of recurrence. *Childs Nerv Syst* 2011, 27(11):1913-8

63. Riggeal BD, Bruce BB, Saindane AM, Ridha MA, Kelly LP, Newman NJ, Biousse V: Clinical course of idiopathic intracranial hypertension with transverse sinus stenosis. *Neurology* 2013, 80(3):289-95

64. Friedman DI: Papilledema and idiopathic intracranial hypertension. *CONTINUUM: Lifelong Learning in Neurology* 2014, 20(4):857-76

65. Yri HM, Wegener M, Sander B, Jensen R: Idiopathic intracranial hypertension is not benign: a long-term outcome study. *Journal of Neurology* 2012, 259(5):886-94

66. Marzoli SB, Ciasca P, Curone M, Cammarata G, Melzi L, Criscuoli A, Bussone G, D'Amico D: Quantitative analysis of optic nerve damage in idiopathic intracranial hypertension (IIH) at diagnosis. *Neurological Sciences* 2013, 34(Suppl 1):S143-S5

67. Chen JJ, Thurtell MJ, Longmuir RA, Garvin MK, Wang JK, Wall M, Kardon RH: Causes and prognosis of visual acuity loss at the time of initial presentation in idiopathic intracranial hypertension. *Invest Ophthalmol Vis Sci* 2015, 56(6):3850-9

68. Bidot S, Clough L, Saindane AM, Newman NJ, Biousse V, Bruce BB: The optic canal size is associated with the severity of papilledema and poor visual function in idiopathic intracranial hypertension. *Journal of Neuro-Ophthalmology* 2016, 36(2):120-5

69. Cello KE, Keltner JL, Johnson CA, Wall M, Nordic Idiopathic Intracranial Hypertension Study Group: Factors affecting visual field outcomes in the Idiopathic Intracranial Hypertension Treatment Trial. *Journal of Neuro-Ophthalmology* 2016, 36(1):6-12

70. Lee YA, Tomsak RL, Sadikovic Z, Bahl R, Sivaswamy L: Use of ocular coherence tomography in children with idiopathic intracranial hypertension - a single center experience *Pediatric Neurology* 2016, 58:101-6

71. Sencer A, Akcakaya MO, Basaran B, Yorukoglu AG, Aydoseli A, Aras Y, Sencan F, Satana B, Aslan I, Unal OF *et al*: Unilateral endoscopic optic nerve decompression for idiopathic intracranial hypertension: a series of 10 patients. *World Neurosurg* 2014, 82(5):745-50

72. Huang LC, Winter TW, Herro AM, Rosa PR, Schiffman JC, Pasol J, Trombly RS, Tawfik M, Lam BL: Ventriculoperitoneal shunt as a treatment of visual loss in idiopathic intracranial hypertension. *Journal of Neuro-Ophthalmology* 2014, 34(3):223-8

73. Obi EE, Lakhani BK, Burns J, Sampath R: Optic nerve sheath fenestration for idiopathic intracranial hypertension: a seven year review of visual outcomes in a tertiary centre. *Clin Neurol Neurosurg* 2015, 137:94-101

74. Gospe SM, 3rd, Bhatti MT, El-Dairi MA: Anatomic and visual function outcomes in paediatric idiopathic intracranial hypertension. *British Journal of Ophthalmology* 2016, 100(4):505-9

75. Vaidya NS, Mahmoud AM, Buzzacco D, Katz SE: Visual outcomes following optic nerve sheath fenestration via the medial transconjunctival approach. *Orbit* 2016, 35(5):271-7

76. Wall M, George D: Idiopathic intracranial hypertension. A prospective study of 50 patients. *Brain* 1991, 114(Pt 1A):155-80

77. Acheson JF, Green WT, Sanders MD: Optic nerve sheath decompression for the treatment of visual failure in chronic raised intracranial pressure. *Journal of Neurology, Neurosurgery & Psychiatry* 1994, 57(11):1426-9

78. Hedges TR, 3rd, Legge RH, Peli E, Yardley CJ: Retinal nerve fiber layer changes and visual field loss in idiopathic intracranial hypertension. *Ophthalmology* 1995, 102(8):1242-7

79. Laemmer R, Heckmann JG, Mardin CY, Schwab S, Laemmer AB: Detection of nerve fiber atrophy in apparently effectively treated papilledema in idiopathic intracranial hypertension. *Graefes Arch Clin Exp Ophthalmol* 2010, 248(12):1787-93

80. Liu IH, Wang AG, Yen MY: Idiopathic intracranial hypertension: clinical features in Chinese patients. *Jpn J Ophthalmol* 2011, 55(2):138-42

81. Ravid S, Shahar E, Schif A, Yehudian S: Visual outcome and recurrence rate in children with idiopathic intracranial hypertension. *Journal of Child Neurology* 2015, 30(11):1448-52

82. Celebisoy N, Ozturk T, Kose T: Rarebit perimetry in the evaluation of visual field defects in idiopathic intracranial hypertension. *Eur J Ophthalmol* 2010, 20(4):756-62

83. Huna-Baron R, Kupersmith MJ: Idiopathic intracranial hypertension in pregnancy. *Journal of Neurology* 2002, 249(8):1078-81

84. Bruce BB, Biousse V, Newman NJ: Update on idiopathic intracranial hypertension. *American Journal of Ophthalmology* 2011, 152(2):163-9

85. Acheson JF: Idiopathic intracranial hypertension and visual function. *Br Med Bull* 2006, 79-80:233-44

86. Botelho PJ, Johnson LN, Arnold AC: The effect of aspirin on the visual outcome of nonarteritic anterior ischemic optic neuropathy. *American Journal of Ophthalmology* 1996, 121(4):450-1

87. Fujimoto N, Adachi-Usami E: Frequency doubling perimetry in resolved optic neuritis. *Invest Ophthalmol Vis Sci* 2000, 41(9):2558-60

88. Genovesi-Ebert F, Di Bartolo E, Lepri A, Poggi V, Romani A, Nardi M: Standardized echography, pattern electroretinography and visual-evoked potential and automated perimetry in the early diagnosis of Graves' neuropathy. *Ophthalmologica* 1998, 212( Suppl 1):101-3

89. Johnson LN, Guy ME, Krohel GB, Madsen RW: Levodopa may improve vision loss in recent-onset, nonarteritic anterior ischemic optic neuropathy. *Ophthalmology* 2000, 107(3):521-6

90. Johnson LN, Gould TJ, Krohel GB: Effect of levodopa and carbidopa on recovery of visual function in patients with nonarteritic anterior ischemic optic neuropathy of longer than six months' duration. *American Journal of Ophthalmology* 1996, 121(1):77-83

91. Bobak SP, Goodwin JA, Guevara RA, Arya A, Grover S: Predictors of visual acuity and the relative afferent pupillary defect in optic neuropathy. *Doc Ophthalmol* 1999, 97(1):81-95

92. Michel O, Oberlander N, Neugebauer P, Neugebauer A, Russmann W: Follow-up of transnasal orbital decompression in severe Graves' ophthalmopathy. *Ophthalmology* 2001, 108(2):400-4

93. Wang J-C, Tow S, Aung T, Lim S-A, Cullen J: The presentation, aeitiology, management and outcome of optic neuritis in an Asian population. *Clin Experiment Ophthalmol* 2001, 29:312-5

94. Hickman SJ, Toosy AT, Jones SJ, Altmann DR, Miszkiel KA, MacManus DG, Barker GJ, Plant GT, Thompson AJ, Miller DH: A serial MRI study following optic nerve mean area in acute optic neuritis. *Brain* 2004, 127(11):2498-505

95. Hickman SJ, Toosy AT, Miszkiel KA, Jones SJ, Altmann DR, MacManus DG, Plant GT, Thompson AJ, Miller DH: Visual recovery following acute optic neuritis--a clinical, electrophysiological and magnetic resonance imaging study. *Journal of Neurology* 2004, 251(8):996-1005

96. Trip SA, Schlottmann PG, Jones SJ, Altmann DR, Garway-Heath DF, Thompson AJ, Plant GT, Miller DH: Retinal nerve fiber layer axonal loss and visual dysfunction in optic neuritis. *Annals of Neurology* 2005, 58(3):383-91

97. Corallo G, Cicinelli S, Papadia M, Bandini F, Uccelli A, Calabria G: Conventional perimetry, short-wavelength automated perimetry, frequency-doubling technology, and visual evoked potentials in the assessment of patients with multiple sclerosis. *Eur J Ophthalmol* 2005, 15(6):730-8

98. Foroozan R, Deramo VA, Buono LM, Jayamanne DG, Sergott RC, Danesh-Meyer H, Savino PJ: Recovery of visual function in patients with biopsy-proven giant cell arteritis. *Ophthalmology* 2003, 110(3):539-42

99. Feldon SE: Computerized expert system for evaluation of automated visual fields from the Ischemic Optic Neuropathy Decompression Trial: methods, baseline fields, and six-month longitudinal follow-up. *Trans Am Ophthalmol Soc* 2004, 102:269-303

100. Wall M, Punke SG, Stickney TL, Brito CF, Withrow KR, Kardon RH: SITA standard in optic neuropathies and hemianopias: a comparison with full threshold testing. *Invest Ophthalmol Vis Sci* 2001, 42(2):528-37

101. Kuprjanowicz L, Goslawski W, Karczewicz D, Szych Z: Evaluation of retinal nerve fiber thickness with scanning laser polarimetry in patients with anterior ischemic optic neuropathy. *Klin Oczna* 2004, 106(Suppl 3 ):440-2

102. Kupersmith MJ, Alban T, Zeiffer B, Lefton D: Contrast-enhanced MRI in acute optic neuritis: Relationship to visual performance. *Brain* 2002, 125(4):812-22

103. Lee EJ, Kim S-J, Choung HK, Kim JH, Yu YS: Incidence and clinical features of ethambutol-induced optic neuropathy in Korea. *Journal of Neuro-Ophthalmology* 2008, 28(4):269-77

104. Kolappan M, Henderson AP, Jenkins TM, Wheeler-Kingshott CA, Plant GT, Thompson AJ, Miller DH: Assessing structure and function of the afferent visual pathway in multiple sclerosis and associated optic neuritis. *Journal of Neurology* 2009, 256(3):305-19

105. Frohman L, Dellatorre K, Turbin R, Bielory L: Clinical characteristics, diagnostic criteria and therapeutic outcomes in autoimmune optic neuropathy. *British Journal of Ophthalmology* 2009, 93(12):1660-6

106. Liao SL, Chang TC, Lin LL: Transcaruncular orbital decompression: an alternate procedure for Graves ophthalmopathy with compressive optic neuropathy. *American Journal of Ophthalmology* 2006, 141(5):810-8

107. Pakrou N, Casson R, Kaines A, Selva D: Multifocal objective perimetry compared with Humphrey full-threshold perimetry in patients with optic neuritis. *Clin Experiment Ophthalmol* 2006, 34(6):562-7

108. Wilhelm B, Ludtke H, Wilhelm H, Braion Study Group: Efficacy and tolerability of 0.2% brimonidine tartrate for the treatment of acute non-arteritic anterior ischemic optic neuropathy (NAION): a 3-month, double-masked, randomised, placebo-controlled trial. *Graefes Arch Clin Exp Ophthalmol* 2006, 244(5):551-8

109. Chang Y-C, Wu W-C, Tsai R-K: Prognosis of Taiwanese patients with isolated optic neuritis after intravenous methylprednisolone pulse therapy. *Journal of the Formosan Medical Association* 2007, 106(8):656-63

110. Bordaberry M, Marques DL, Pereira-Lima JC, Marcon IM, Schmid H: Repeated peribulbar injections of triamcinolone acetonide: a successful and safe treatment for moderate to severe Graves' ophthalmopathy. *Acta Ophthalmol (Oxf)* 2009, 87(1):58-64

111. Laron M, Cheng H, Zhang B, Schiffman JS, Tang RA, Frishman LJ: Comparison of multifocal visual evoked potential, standard automated perimetry and optical coherence tomography in assessing visual pathway in multiple sclerosis patients. *Mult Scler* 2010, 16(4):412-26

112. Trip SA, Schlottmann PG, Jones SJ, Kallis C, Altmann DR, Garway-Heath DF, Thompson AJ, Plant GT, Miller DH: Scanning laser polarimetry quantification of retinal nerve fiber layer thinning following optic neuritis. *Journal of Neuro-Ophthalmology* 2010, 30(3):235-42

113. Trip SA, Schlottmann PG, Jones SJ, Garway-Heath DF, Thompson AJ, Plant GT, Miller DH: Quantification of optic nerve head topography in optic neuritis: a pilot study. *British Journal of Ophthalmology* 2006, 90(9):1128-31

114. Costello F, Hodge W, Pan YI, Eggenberger E, Freedman MS: Using retinal architecture to help characterize multiple sclerosis patients. *Canadian Journal of Ophthalmology* 2010, 45(5):520-6

115. Serbecic N, Aboul-Enein F, Beutelspacher SC, Graf M, Kircher K, Geitzenauer W, Brannath W, Lang P, Kristoferitsch W, Lassmann H *et al*: Heterogeneous pattern of retinal nerve fiber layer in multiple sclerosis. High resolution optical coherence tomography: potential and limitations. *PLoS One* 2010, 5(11) DOI: 10.1371/journal.pone.0013877.

116. Labonia AF, Carnovale-Scalzo G, Paola A, De' Morelli G, Scorcia V, Bruzzichessi D, Scorcia G, Costante G: Subclinical visual field alterations are commonly present in patients with Graves' Orbitopathy and are mainly related to the clinical activity of the disease. *Exp Clin Endocrinol Diabetes* 2008, 116(6):347-51

117. Cheng H, Laron M, Schiffman JS, Tang RA, Frishman LJ: The relationship between visual field and retinal nerve fiber layer measurements in patients with multiple sclerosis. *Invest Ophthalmol Vis Sci* 2007, 48(12):5798-805

118. Bellusci C, Savini G, Carbonelli M, Carelli V, Sadun AA, Barboni P: Retinal nerve fiber layer thickness in nonarteritic anterior ischemic optic neuropathy: OCT characterization of the acute and resolving phases. *Graefes Arch Clin Exp Ophthalmol* 2008, 246(5):641-7

119. Hood DC, Anderson S, Rouleau J, Wenick AS, Grover LK, Behrens MM, Odel JG, Lee AG, Kardon RH: Retinal nerve fiber structure versus visual field function in patients with ischemic optic neuropathy. A test of a linear model. *Ophthalmology* 2008, 115(5):904-10

120. Almarcegui C, Dolz I, Pueyo V, Garcia E, Fernandez FJ, Martin J, Ara JR, Honrubia F: Correlation between functional and structural assessments of the optic nerve and retina in multiple sclerosis patients. *Neurophysiol Clin* 2010, 40(3):129-35

121. Scherer RW, Feldon SE, Levin L, Langenberg P, Katz J, Keyl PM, Wilson PD, Kelman SE, Dickersin K, Ischemic Optic Neuropathy Decompression Trial Research Group: Visual fields at follow-up in the Ischemic Optic Neuropathy Decompression Trial: evaluation of change in pattern defect and severity over time. *Ophthalmology* 2008, 115(10):1809-17

122. Noval S, Contreras I, Rebolleda G, Munoz-Negrete FJ: Optical coherence tomography versus automated perimetry for follow-up of optic neuritis. *Acta Ophthalmol Scand* 2006, 84(6):790-4

123. Contreras I, Noval S, Rebolleda G, Munoz-Negrete FJ: Follow-up of nonarteritic anterior ischemic optic neuropathy with optical coherence tomography. *Ophthalmology* 2007, 114(12):2338-44

124. Deleon-Ortega J, Carroll KE, Arthur SN, Girkin CA: Correlations between retinal nerve fiber layer and visual field in eyes with nonarteritic anterior ischemic optic neuropathy. *American Journal of Ophthalmology* 2007, 143(2):288-94

125. Jung CS, Bruce B, Newman NJ, Biousse V: Visual function in anterior ischemic optic neuropathy: effect of Vision Restoration Therapy--a pilot study. *J Neurol Sci* 2008, 268:145-9

126. Falavarjani KG, Sanjari MS, Modarres M, Aghamohammadi F: Clinical profile of patients with nonarteritic anterior ischemic optic neuropathy presented to a referral center from 2003 to 2008. *Arch Iran Med* 2009, 12(5):472-7

127. Kho RC, Al-Obailan M, Arnold AC: Bitemporal visual field defects in ethambutol-induced optic neuropathy. *Journal of Neuro-Ophthalmology* 2011, 31(2):121-6

128. Kitsos G, Detorakis ET, Papakonstantinou S, Kyritsis AP, Pelidou SH: Perimetric and peri‐papillary nerve fibre layer thickness findings in multiple sclerosis. *European Journal of Neurology* 2011, 18(5):719-25

129. Connick P, Kolappan M, Crawley C, Webber DJ, Patani R, Michell AW, Du M-Q, Luan S-L, Altmann DR, Thompson AJ *et al*: Autologous mesenchymal stem cells for the treatment of secondary progressive multiple sclerosis: an open-label phase 2a proof-of-concept study. *Lancet Neurology* 2012, 11(2):150-6

130. Jeon C, Shin JH, Woo KI, Kim YD: Clinical profile and visual outcomes after treatment in patients with dysthyroid optic neuropathy. *Korean J Ophthalmol* 2012, 26(2):73-9

131. Lee J-Y, Cho K, Park K-A, Oh SY: Analysis of retinal layer thicknesses and their clinical correlation in patients with traumatic optic neuropathy. *PLoS One* 2016, 11(6) DOI: 10.1371.journal.pone.0157388.

132. Hata M, Oishi A, Muraoka Y, Miyamoto K, Kawai K, Yokota S, Fujimoto M, Miyata M, Yoshimura N: Structural and Functional Analyses in Nonarteritic Anterior Ischemic Optic Neuropathy: Optical Coherence Tomography Angiography Study. *Journal of Neuro-Ophthalmology* 2017, 37(2):140-8

133. Lu P, Sha Y, Wan H, Wang F, Tian G, Tang W: Assessment of nonarteritic anterior ischemic optic neuropathy with intravoxel incoherent motion diffusion-weighted imaging using readout-segmented echo-planar imaging, parallel imaging, and 2D navigator-based reacquisition. *Journal of Magnetic Resonance Imaging* 2017, 46(6):1760-6

134. Stiebel-Kalish H, Lotan I, Brody J, Chodick G, Bialer O, Marignier R, Bach M, Hellmann MA: Retinal nerve fibre layer may be better preserved in MOG-IgG versus AQP4-IgG optic neuritis: a cohort study. *PLoS One* 2017, 12(1) DOI: 10.1371/journal.pone.0170847.

135. Choi JC, Oropesa S, Callahan AB, Glass LR, Teo L, Cestari DM, Kazim M, Freitag SK: Patterns of visual field changes in thyroid eye disease. *Orbit* 2017, 36(4):201-7

136. Urano T, Matsuura T, Yukawa E, Arai M, Hara Y, Yamakawa R: Retinal nerve fiber layer thickness changes following optic neuritis caused by multiple sclerosis. *Jpn J Ophthalmol* 2011, 55(1):45-8

137. Gallo A, Esposito F, Sacco R, Docimo R, Bisecco A, Della Corte M, D'Ambrosio A, Corbo D, Rosa N, Lanza M *et al*: Visual resting-state network in relapsing-remitting MS with and without previous optic neuritis. *Neurology* 2012, 79(14):1458-65

138. Costello F, Pan YI, Yeh EA, Hodge W, Burton JM, Kardon R: The temporal evolution of structural and functional measures after acute optic neuritis. *Journal of Neurology, Neurosurgery & Psychiatry* 2015, 86(12):1369-73

139. Narayanan D, Cheng H, Tang RA, Frishman LJ: Longitudinal evaluation of visual function in multiple sclerosis. *Optometry and Vision Science* 2015, 92(10):976-85

140. Lee TH, Ji YS, Park SW, Heo H: Retinal ganglion cell and axonal loss in optic neuritis: risk factors and visual functions. *Eye* 2017, 31(3):467-74

141. Garcia-Martin E, Pueyo V, Almarcegui C, Martin J, Ara JR, Sancho E, Pablo LE, Dolz I, Fernandez J: Risk factors for progressive axonal degeneration of the retinal nerve fibre layer in multiple sclerosis patients. *British Journal of Ophthalmology* 2011, 95(11):1577-82

142. Garcia-Martin E, Rodriguez-Mena D, Herrero R, Almarcegui C, Dolz I, Martin J, Ara JR, Larrosa JM, Polo V, Fernández J *et al*: Neuro-ophthalmologic evaluation, quality of life, and functional disability in patients with MS. *Neurology* 2013, 81(1):76-83

143. Alpay A, Guney T, Unal A, Ugurbas SH: Comparison of retinal nerve fibre layer thickness with visual evoked potential and visual field in patients with multiple sclerosis. *Clin Experiment Ophthalmol* 2012, 40(1):e25-31 DOI: 10.1111/j.1442-9071.2011.02619.x

144. Rodriguez-Mena D, Almarcegui C, Dolz I, Herrero R, Bambo MP, Fernandez J, Pablo LE, Garcia-Martin E: Electropysiologic evaluation of the visual pathway in patients with multiple sclerosis. *Journal of Clinical Neurophysiology* 2013, 30(4):376-81

145. Prokosch V, Thanos S: Visual outcome of patients following NAION after treatment with adjunctive fluocortolone. *Restorative Neurology and Neuroscience* 2014, 32(3):381-9

146. Karahan E, Karti O, Koskderelioglu A, Karti DT, Uyar M, Kale MY, Gedizlioglu M: Pupil cycle time: as indicator of visual pathway dysfunction in multiple sclerosis. *Acta Neurol Belg* 2017, 117(1):75-81

147. Matsumoto Y, Mori S, Ueda K, Kurimoto T, Kanamori A, Yamada Y, Nakashima I, Nakamura M: Impact of the anti-aquaporin-4 autoantibody on inner retinal structure, function and structure-function associations in Japanese patients with optic neuritis. *PLoS One* 2017, 12(2) DOI: 10.1371/journal.pone.0171880.

148. Bilgin S, Sami İlker S, Çavdar E, Türker İ: Subklinik Multipl Skleroz Hastalarının Desen Görsel Uyarılmış Potansiyeller ve Görme Alanı Testi ile Değerlendirilmesi. *Turkish Journal of Ophthalmology / Turk Oftalmoloji Dergisi* 2014, 44(3):207-11 DOI: 10.4274/tjo.75983.

149. Curro N, Covelli D, Vannucchi G, Campi I, Pirola G, Simonetta S, Dazzi D, Guastella C, Pignataro L, Beck-Peccoz P *et al*: Therapeutic outcomes of high-dose intravenous steroids in the treatment of dysthyroid optic neuropathy. *Thyroid* 2014, 24(5):897-905

150. Choe CH, Cho RI, Elner VM: Comparison of lateral and medial orbital decompression for the treatment of compressive optic neuropathy in thyroid eye disease. *Ophthal Plast Reconstr Surg* 2011, 27(1):4-11

151. Bisaga GN, Kovalenko AV, Kovalenko I: Diagnosis and differential diagnosis of demyelinating optic neuropathy in multiple sclerosis. *Zh Nevrol Psikhiatr Im S S Korsakova* 2012, 112(9 Pt 2):10-22

152. Ben Simon GJ, Katz G, Zloto O, Leiba H, Hadas B, Huna-Baron R: Age differences in clinical manifestation and prognosis of thyroid eye disease. *Graefes Arch Clin Exp Ophthalmol* 2015, 253(12):2301-8

153. Jiang L, Chen L, Qiu X, Jiang R, Wang Y, Xu L, Lai TYY: Choroidal thickness in Chinese patients with non-arteritic anterior ischemic optic neuropathy. *BMC Ophthalmology* 2016, 16(153) DOI: 10.1186/s12886-016-0313-2.

154. Esfahani MR, Harandi ZA, Kiumehr S, Gholmi A, Tabasi A, Piri N, Mirshahi A, Ahmadabadi MN, Movassat M, Fakhraee G: Memantine treatment in acute nonarteritic anterior ischemic optic neuropathy: a randomized clinical trial. *Iranian Journal of Ophthalmology* 2011, 23(1):11-20

155. Rebolleda G, Perez-Lopez M, Casas LP, Contreras I, Munoz-Negrete FJ: Visual and anatomical outcomes of non-arteritic anterior ischemic optic neuropathy with high-dose systemic corticosteroids. *Graefes Arch Clin Exp Ophthalmol* 2013, 251(1):255-60

156. De Dompablo E, Garcia-Montesinos J, Munoz-Negrete FJ, Rebolleda G: Ganglion cell analysis at acute episode of nonarteritic anterior ischemic optic neuropathy to predict irreversible damage: a prospective study. *Graefes Arch Clin Exp Ophthalmol* 2016, 254(9):1793-800

157. Modarres M, Falavarjani KG, Nazari H, Sanjari MS, Aghamohammadi F, Homaii M, Samiy N: Intravitreal erythropoietin injection for the treatment of non-arteritic anterior ischaemic optic neuropathy. *British Journal of Ophthalmology* 2011, 95(7):992-5

158. Moghimi S, Vahedian Z, Amini H, Soleimani M, Eslami Y, Fakhraie G, Zarei R: Correlation between retinal nerve fiber layer thickness measured by GDx and visual field in nonarteritic anterior ischemic optic neuropathy: a comparison with the contralateral normal eye. *Iranian Journal of Ophthalmology* 2011, 23(2):35-43

159. Monteiro ML, Fernandes DB, Apostolos-Pereira SL, Callegaro D: Quantification of retinal neural loss in patients with neuromyelitis optica and multiple sclerosis with or without optic neuritis using Fourier-domain optical coherence tomography. *Invest Ophthalmol Vis Sci* 2012, 53(7):3959-66

160. Papchenko T, Grainger BT, Savino PJ, Gamble GD, Danesh-Meyer HV: Macular thickness predictive of visual field sensitivity in ischaemic optic neuropathy. *Acta Ophthalmol (Oxf)* 2012, 90(6):e463-e9

161. Hokazono K, Raza AS, Oyamada MK, Hood DC, Monteiro ML: Pattern electroretinogram in neuromyelitis optica and multiple sclerosis with or without optic neuritis and its correlation with FD-OCT and perimetry. *Doc Ophthalmol* 2013, 127(3):201-15

162. Kupersmith MJ, Anderson S, Durbin M, Kardon R: Scanning laser polarimetry, but not optical coherence tomography predicts permanent visual field loss in acute nonarteritic anterior ischemic optic neuropathy. *Invest Ophthalmol Vis Sci* 2013, 54(8):5514-9

163. Rootman DB, Gill HS, Margolin EA: Intravitreal bevacizumab for the treatment of nonarteritic anterior ischemic optic neuropathy: a prospective trial. *Eye* 2013, 27(4):538-44

164. Saxena R, Bandyopadhyay G, Singh D, Singh S, Sharma P, Menon V: Evaluation of changes in retinal nerve fiber layer thickness and visual functions in cases of optic neuritis and multiple sclerosis. *Indian Journal of Ophthalmology* 2013, 61(10):562-6

165. Martinez-Lapiscina EH, Fraga-Pumar E, Gabilondo I, Martinez-Heras E, Torres-Torres R, Ortiz-Perez S, Llufriu S, Tercero A, Andorra M, Roca MF *et al*: The multiple sclerosis visual pathway cohort: understanding neurodegeneration in MS. *BMC Res Notes* 2014, 7(910) DOI: 10.1186/1756-0500-7-910.

166. Cennamo G, Romano MR, Vecchio EC, Minervino C, Della Guardia C, Velotti N, Carotenuto A, Montella S, Orefice G, Cennamo G: Anatomical and functional retinal changes in multiple sclerosis. *Eye* 2016, 30(3):456-62

167. Longbrake EE, Lancia S, Tutlam N, Trinkaus K, Naismith RT: Quantitative visual tests after poorly recovered optic neuritis due to multiple sclerosis. *Mult Scler Relat Disord* 2016, 10:198-203

168. Khalil DH, Said MM, Abdelhakim MASE, Labeeb DM: OCT and visual field changes as useful markers for follow-up of axonal loss in multiple sclerosis in Egyptian patients. *Ocular Immunology & Inflammation* 2017, 25(3):315-22

169. Lyttle DP, Johnson LN, Margolin EA, Madsen RW: Levodopa as a possible treatment of visual loss in nonarteritic anterior ischemic optic neuropathy. *Graefes Arch Clin Exp Ophthalmol* 2016, 254(4):757-64

170. Esfahani MR, Harandi ZA, Movasat M, Nikdel M, Adelpour M, Momeni A, Merat H, Fard MA: Memantine for axonal loss of optic neuritis. *Graefes Arch Clin Exp Ophthalmol* 2012, 250(6):863-9

171. Carter KD, Frueh BR, Hessburg TP, Musch DC: Long-term efficacy of orbital decompression for compressive optic neuropathy of Graves' eye disease. *Ophthalmology* 1991, 98(9):1435-42

172. McDonald WI, Barnes D: The ocular manifestations of multiple sclerosis. 1. Abnormalities of the afferent visual system. *Journal of Neurology, Neurosurgery & Psychiatry* 1992, 55(9):747-52

173. Davis FA, Stefoski D, Rush J: Orally administered 4-aminopyridine improves clinical signs in multiple sclerosis. *Annals of Neurology* 1990, 27(2):186-92

174. Gerling J, Meyer JH, Kommerell G: Visual field defects in optic neuritis and anterior ischemic optic neuropathy: distinctive features. *Graefes Arch Clin Exp Ophthalmol* 1998, 236(3):188-92

175. Hayreh SS, Zimmerman B, Kardon RH: Visual improvement with corticosteroid therapy in giant cell arteritis. Report of a large study and review of literature. *Acta Ophthalmol Scand* 2002, 80(4):355-67

176. Korinth MC, Ince A, Banghard W, Gilsbach JM: Follow-up of extended pterional orbital decompression in severe Graves' ophthalmopathy. *Acta Neurochir (Wien)* 2002, 144(2):113-20

177. Cruz AA, Leme VR: Orbital decompression: a comparison between trans-fornix/transcaruncular inferomedial and coronal inferomedial plus lateral approaches. *Ophthal Plast Reconstr Surg* 2003, 19(6):440-5

178. Bee YS, Lin MC, Wang CC, Sheu SJ: Optic neuritis: clinical analysis of 27 cases. *Kaohsiung J Med Sci* 2003, 19(3):105-12

179. Soares-Welch CV, Fatourechi V, Bartley GB, Beatty CW, Gorman CA, Bahn RS, Bergstralh EJ, Schleck CD, Garrity JA: Optic neuropathy of Graves disease: results of transantral orbital decompression and long-term follow-up in 215 patients. *American Journal of Ophthalmology* 2003, 136(3):433-41

180. Hayreh SS: Posterior ischaemic optic neuropathy: clinical features, pathogenesis, and management. *Eye* 2004, 18(11):1188-206

181. Vidovic T, Cerovski B, Vidovic DH, Cerovski J, Novak-Laus K: Inapparent visual field defects in multiple sclerosis patients. *Coll Antropol* 2005, 29(Suppl 1):67-73

182. Carta A, Ferrigno L, Leaci R, Kosmarikou A, Zola E, Gomarasca S: Long-term outcome after conservative treatment of indirect traumatic optic neuropathy. *Eur J Ophthalmol* 2006, 16(6):847-50

183. Hayreh SS, Zimmerman MB: Non-arteritic anterior ischemic optic neuropathy: role of systemic corticosteroid therapy. *Graefes Arch Clin Exp Ophthalmol* 2008, 246(7):1029-46

184. Hayreh SS, Zimmerman MB: Optic disc edema in non-arteritic anterior ischemic optic neuropathy. *Graefes Arch Clin Exp Ophthalmol* 2007, 245(8):1107-21

185. Menon V, Jain D, Saxena R, Sood R: Prospective evaluation of visual function for early detection of ethambutol toxicity. *British Journal of Ophthalmology* 2009, 93(9):1251-4

186. Hayreh SS: Management of ischemic optic neuropathies. *Indian Journal of Ophthalmology* 2011, 59(2):123-36

187. Cullen JF, Chung SH: Non-arteritic anterior ischaemic optic neuropathy (NA-AION): outcome for visual acuity and visual field defects, the Singapore scene 2. *Singapore Medical Journal* 2012, 53(2):88-90

188. Mercado JL, Purvin VA, Kawasaki A, Wudunn D: Bilateral sequential nonarteritic anterior ischemic optic neuropathy: a comparison of visual outcomes in fellow eyes using quantitative analysis of goldmann visual fields. *Archives of Ophthalmology* 2012, 130(7):863-7

189. Hayreh SS, Zimmerman MB: Bilateral nonarteritic anterior ischemic optic neuropathy: comparison of visual outcome in the two eyes. *Journal of Neuro-Ophthalmology* 2013, 33(4):338-43

190. Fukuchi M, Kishi S, Li D, Akiyama H: Acute ganglion cell loss during rapid visual recovery in optic neuritis. *Graefes Arch Clin Exp Ophthalmol* 2016, 254(12):2355-60

191. Graf M, Meienberg O: Octopus perimetry in neuro-ophthalmologic diseases. A contribution to the problem of optimal program choice based on 427 cases. *Klinische Monatsblatter fur Augenheilkunde* 1991, 198(6):530-7

192. Haas A, Walzl M, Jesenik F, Walzl B, Berghold A, Bergloff J, Feigl B, Faulborn J: Application of HELP in nonarteritic anterior ischemic optic neuropathy: a prospective, randomized, controlled study. *Graefes Arch Clin Exp Ophthalmol* 1997, 235(1):14-9

193. Nizankowska MH, Turno-Krecicka A, Misiuk-Hojlo M, Ejma M, Batycka-Ugorska I: Early diagnosis of optic nerve neuropathy in multiple sclerosis. *Klin Oczna* 1996, 98(1):21-5

194. Nevalainen J, Krapp E, Paetzold J, Mildenberger I, Besch D, Vonthein R, Keltner JL, Johnson CA, Schiefer U: Visual field defects in acute optic neuritis--distribution of different types of defect pattern, assessed with threshold-related supraliminal perimetry, ensuring high spatial resolution. *Graefes Arch Clin Exp Ophthalmol* 2008, 246(4):599-607

195. Wang RY, Zhong Y, Dong FT, Zhao P, Shi W, Song DL: [Application of Octopus 101 automated perimetry with kinetic and static program in patients with the anterior ischemic optic neuropathy]. *Chung-Hua i Hsueh Tsa Chih* 2010, 90(19):1322-5

196. Kernstock C, Beisse F, Wiethoff S, Mast A, Krapp E, Grund R, Dietzsch J, Lagreze W, Fischer D, Schiefer U: Assessment of functional and morphometric endpoints in patients with non-arteritic anterior ischemic optic neuropathy (NAION). *Graefes Arch Clin Exp Ophthalmol* 2014, 252(3):515-21

197. Vidovic T, Cerovski B, Peric S, Kordic R, Mrazovac D: Corticosteroid therapy in patients with non-arteritic anterior ischemic optic neuropathy. *Coll Antropol* 2015, 39(1):63-6

198. Atipo-Tsiba PW, Borruat FX: Traumatic dysfunction of the optic chiasm. *Klinische Monatsblatter fur Augenheilkunde* 2003, 220(3):138-41

199. Wall M: High-pass resolution perimetry in optic neuritis. *Invest Ophthalmol Vis Sci* 1991, 32(9):2525-9

200. Lycke J, Tollesson PO, Frisen L: Asymptomatic visual loss in multiple sclerosis. *Journal of Neurology* 2001, 248(12):1079-86

201. Frisén L: Performance of a rapid rarebit central-vision test with optic neuropathies. *Optometry & Vision Science* 2012, 89(8):1192-5

202. Edgar GK, Foster DH, Honan WP, Heron JR, Snelgar RS: Optic neuritis: variations in temporal modulation sensitivity with retinal eccentricity. *Brain* 1990, 113(Pt 2):487-96

203. Honan WP, Heron JR, Foster DH, Edgar GK, Scase MO, Collins MF: Visual loss in multiple sclerosis and its relation to previous optic neuritis, disease duration and clinical classification. *Brain* 1990, 113(Pt 4):975-87

204. Wilhelm H, Meilinger S, Apfelstedt E: [Relation between relative afferent pupillary defect and suprathreshold automated perimetry]. *Klinische Monatsblatter fur Augenheilkunde* 1997, 210(6):365-9

205. Radoi C, Garcia T, Brugniart C, Ducasse A, Arndt C: Intravitreal triamcinolone injections in non-arteritic anterior ischemic optic neuropathy. *Graefes Arch Clin Exp Ophthalmol* 2014, 252(2):339-45

206. Hattenhauer MG, Leavitt JA, Hodge DO, Grill R, Gray DT: Incidence of nonarteritic anterior ischemic optic neuropathy. *American Journal of Ophthalmology* 1997, 123(1):103-7

207. DelMonte DW, Bhatti MT: Ischemic optic neuropathy. *International Ophthalmology Clinics* 2009, 49(3):35-62

208. Ismail S, Wan Hazabbah WH, Muhd-Nor NI, Daud J, Embong Z: Clinical profile and aetiology of optic neuritis in Hospital Universiti Sains Malaysia--5 years review. *Med J Malaysia* 2012, 67(2):159-64

209. Biousse V, Newman NJ: Diagnosis and clinical features of common optic neuropathies. *Lancet Neurology* 2016, 15(13):1355-67

210. Choi SY, Hwang JM: Optic neuropathy associated with ethambutol in Koreans. *Korean J Ophthalmol* 1997, 11(2):106-10

211. Armstrong RA: Multiple sclerosis and the eye. *Ophthalmic Physiol Opt* 1999, 19 (Suppl 2):S32-42

212. Spoor TC, McHenry JG: Long-term effectiveness of optic nerve sheath decompression for pseudotumor cerebri. *Archives of Ophthalmology* 1993, 111(5):632-5

213. Rowe F, Thompson C, Webster A: Incidence of bitemporal hemianopic visual field defects in pituitary tumours. In: *International Orthoptic Congress: 1995; Kyoto, Japan*; 1995: 279-83.

214. Wall M, Conway MD, House PH, Allely R: Evaluation of sensitivity and specificity of spatial resolution and Humphrey automated perimetry in pseudotumor cerebri patients and normal subjects. *Invest Ophthalmol Vis Sci* 1991, 32(13):3306-12

215. Phillips PH, Repka MX, Lambert SR: Pseudotumor cerebri in children. *J Aapos* 1998, 2(1):33-8

216. Kerrison JB, Lynn MJ, Baer CA, Newman SA, Biousse V, Newman NJ: Stages of improvement in visual fields after pituitary tumor resection. *American Journal of Ophthalmology* 2000, 130(6):813-20

217. Rowe FJ: Visual disturbance in chiasmal lesions. *British Orthoptic Journal* 1996, 53:1-9

218. Chen C, Okera S, Davies PE, Selva D, Crompton JL: Craniopharyngioma: a review of long-term visual outcome. *Clin Experiment Ophthalmol* 2003, 31(3):220-8

219. Baldeweg SE, Pollock JR, Powell M, Ahlquist J: A spectrum of behaviour in silent corticotroph pituitary adenomas. *Br J Neurosurg* 2005, 19(1):38-42

220. Fujimoto N, Saeki N, Miyauchi O, Adachi-Usami E: Criteria for early detection of temporal hemianopia in asymptomatic pituitary tumor. *Eye* 2002, 16(6):731-8

221. Wall M, Neahring RK, Woodward KR: Sensitivity and specificity of frequency doubling perimetry in neuro-ophthalmic disorders: a comparison with conventional automated perimetry. *Invest Ophthalmol Vis Sci* 2002, 43(4):1277-83

222. Gnanalingham KK, Bhattacharjee S, Pennington R, Ng J, Mendoza N: The time course of visual field recovery following transphenoidal surgery for pituitary adenomas: predictive factors for a good outcome. *Journal of Neurology, Neurosurgery & Psychiatry* 2005, 76(3):415-9

223. Thomas R, Shenoy K, Seshadri MS, Muliyil J, Rao A, Paul P: Visual field defects in non-functioning pituitary adenomas. *Indian Journal of Ophthalmology* 2002, 50(2):127-30

224. Foroozan R: Chiasmal syndromes. *Curr Opin Ophthalmol* 2003, 14(6):325-31

225. Pereira A, Monteiro ML: [Computerized and manual perimetry in patients with severe temporal visual field defects due to suprasellar tumors]. *Arq Bras Oftalmol* 2005, 68(5):587-91

226. Alleyne CH, Jr., Barrow DL, Oyesiku NM: Combined transsphenoidal and pterional craniotomy approach to giant pituitary tumors. *Surgical Neurology* 2002, 57(6):380-90

227. Dekkers OM, Pereira AM, Roelfsema F, Voormolen JH, Neelis KJ, Schroijen MA, Smit JW, Romijn JA: Observation alone after transsphenoidal surgery for nonfunctioning pituitary macroadenoma. *J Clin Endocrinol Metab* 2006, 91(5):1796-801

228. Margalit N, Kesler A, Ezer H, Freedman S, Ram Z: Tuberculum and diaphragma sella meningioma--surgical technique and visual outcome in a series of 20 cases operated over a 2.5-year period. *Acta Neurochir (Wien)* 2007, 149(12):1199-204

229. Gedik S, Gur S, Atalay B, Colak M, Altinors N, Akova YA: Humphrey visual field analysis, visual field defects, and ophthalmic findings in patients with macro pituitary adenoma. *Saudi Med J* 2007, 28(9):1380-4

230. Zhong Y, Shen X, Min Y, Cheng Y, Jiao Q: The role of blue-on-yellow perimetry in patients with pituitary tumor. *Annals Of Ophthalmology* 2009, 41(1):40-3

231. Chandrasekaran S, McCluskey P, Minassian D, Assaad N: Visual outcomes for optic nerve sheath fenestration in pseudotumour cerebri and related conditions. *Clin Experiment Ophthalmol* 2006, 34(7):661-5

232. Monteiro ML, Moura FC, Cunha LP: Frequency doubling perimetry in patients with mild and moderate pituitary tumor-associated visual field defects detected by conventional perimetry. *Arq Bras Oftalmol* 2007, 70(2):323-9

233. Piekarska A, Lubinski W, Goslawski W, Wieliczko W, Syrenicz A, Olszowski T, Karczewicz D: [Value of mfVEP test in pituitary tumors diagnosis]. *Klin Oczna* 2008, 110(7-9):247-51

234. Moura FC, Costa-Cunha LV, Malta RF, Monteiro ML: Relationship between visual field sensitivity loss and quadrantic macular thickness measured with Stratus-Optical coherence tomography in patients with chiasmal syndrome. *Arq Bras Oftalmol* 2010, 73(5):409-13

235. Danesh-Meyer HV, Papchenko T, Savino PJ, Law A, Evans J, Gamble GD: In vivo retinal nerve fiber layer thickness measured by optical coherence tomography predicts visual recovery after surgery for parachiasmal tumors. *Invest Ophthalmol Vis Sci* 2008, 49(5):1879-85

236. Danesh-Meyer HV, Carroll SC, Foroozan R, Savino PJ, Fan J, Jiang Y, Vander Hoorn S: Relationship between retinal nerve fiber layer and visual field sensitivity as measured by optical coherence tomography in chiasmal compression. *Invest Ophthalmol Vis Sci* 2006, 47(11):4827-35

237. Danesh-Meyer HV, Carroll SC, Gaskin BJ, Gao A, Gamble GD: Correlation of the multifocal visual evoked potential and standard automated perimetry in compressive optic neuropathies. *Invest Ophthalmol Vis Sci* 2006, 47(4):1458-63

238. Noval S, Contreras I, Rebolleda G, Munoz-Negrete FJ, Ruiz de Zarate B: A comparison between Humphrey and frequency doubling perimetry for chiasmal visual field defects. *Eur J Ophthalmol* 2005, 15(6):739-45

239. Cunha LP, Oyamada MK, Monteiro ML: Pattern electroretinograms for the detection of neural loss in patients with permanent temporal visual field defect from chiasmal compression. *Doc Ophthalmol* 2008, 117(3):223-32

240. Monteiro ML, Costa-Cunha LV, Cunha LP, Malta RF: Correlation between macular and retinal nerve fibre layer Fourier-domain OCT measurements and visual field loss in chiasmal compression. *Eye* 2010, 24(8):1382-90

241. Monteiro ML, Zambon BK, Cunha LP: Predictive factors for the development of visual loss in patients with pituitary macroadenomas and for visual recovery after optic pathway decompression. *Canadian Journal of Ophthalmology* 2010, 45(4):404-8

242. Monteiro ML, Cunha LP, Costa-Cunha LV, Maia OO, Jr., Oyamada MK: Relationship between optical coherence tomography, pattern electroretinogram and automated perimetry in eyes with temporal hemianopia from chiasmal compression. *Invest Ophthalmol Vis Sci* 2009, 50(8):3535-41

243. Dutta P, Gyurmey T, Bansal R, Pathak A, Dhandapani S, Rai A, Bhansali A, Kumar Mukherjee K, Mukherjee KK: Visual outcome in 2000 eyes following microscopic transsphenoidal surgery for pituitary adenomas: protracted blindness should not be a deterrent. *Neurology India* 2016, 64(6):1247-53

244. Luomaranta T, Raappana A, Saarela V, Liinamaa MJ: Factors affecting the visual outcome of pituitary adenoma patients treated with endoscopic transsphenoidal surgery. *World Neurosurg* 2017, 105:422-31

245. Dhasmana R, Nagpal RC, Sharma R, Bansal KK, Bahadur H: Visual fields at presentation and after trans-sphenoidal resection of pituitary adenomas. *Journal of Ophthalmic & Vision Research* 2011, 6(3):187-91

246. Musluman AM, Cansever T, Yilmaz A, Kanat A, Oba E, Cavusoglu H, Sirinoglu D, Aydin Y: Surgical results of large and giant pituitary adenomas with special consideration of ophthalmologic outcomes. *World Neurosurg* 2011, 76(1-2):141-8

247. Zhang L, Sun C, Sun X: [The clinical features and value of macular ganglion cell complex thickness patterns in patients with optic chiasma lesion]. *Chung-Hua Yen Ko Tsa Chih* 2016, 52(5):335-42

248. Barzaghi LR, Medone M, Losa M, Bianchi S, Giovanelli M, Mortini P: Prognostic factors of visual field improvement after trans-sphenoidal approach for pituitary macroadenomas: review of the literature and analysis by quantitative method. *Neurosurg Rev* 2012, 35(3):369-78

249. Mortini P, Barzaghi LR, Serra C, Orlandi V, Bianchi S, Losa M: Visual outcome after fronto-temporo-orbito-zygomatic approach combined with early extradural and intradural optic nerve decompression in tuberculum and diaphragma sellae meningiomas. *Clin Neurol Neurosurg* 2012, 114(6):597-606

250. Lee IH, Miller NR, Zan E, Tavares F, Blitz AM, Sung H, Yousem DM, Boland MV: Visual defecs in patients with pituitary adenomas: the myth of bitmeporal hemianopsia. *American Journal of Roentgenology* 2015, 205(5):W512-8

251. Qiao N, Ye Z, Shou X, Wang Y, Li S, Wang M, Zhao Y: Discrepancy between structural and functional visual recovery in patients after trans-sphenoidal pituitary adenoma resection. *Clin Neurol Neurosurg* 2016, 151:9-17

252. Qiao N, Zhang Y, Ye Z, Shen M, Shou X, Wang Y, Li S, Wang M, Zhao Y: Comparison of multifocal visual evoked potential, static automated perimetry, and optical coherence tomography findings for assessing visual pathways in patients with pituitary adenomas. *Pituitary* 2015, 18(5):598-603

253. Bialer OY, Goldenberg-Cohen N, Toledano H, Snir M, Michowiz S: Retinal NFL thinning on OCT correlates with visual field loss in pediatric craniopharyngioma. *Canadian Journal of Ophthalmology* 2013, 48(6):494-9

254. Kan E, Kan EK, Atmaca A, Atmaca H, Colak R: Visual field defects in 23 acromegalic patients. *Int Ophthalmol* 2013, 33(5):521-5

255. Tieger MG, Hedges TR, 3rd, Ho J, Erlich-Malona NK, Vuong LN, Athappilly GK, Mendoza-Santiesteban CE: Ganglion Cell Complex Loss in Chiasmal Compression by Brain Tumors. *Journal of Neuro-Ophthalmology* 2017, 37(1):7-12

256. Ogra S, Nichols AD, Stylli S, Kaye AH, Savino PJ, Danesh-Meyer HV: Visual acuity and pattern of visual field loss at presentation in pituitary adenoma. *Journal of Clinical Neuroscience* 2014, 21(5):735-40

257. Yum HR, Park SH, Park HY, Shin SY: Macular ganglion cell analysis determined by Cirrus HD optical coherence tomography for early detecting chiasmal compression. *PLoS One* 2016, 11(4) DOI: 10.1371/journal.pone.0153064.

258. Lee JP, Park IW, Chung YS: The volume of tumor mass and visual field defect in patients with pituitary macroadenoma. *Korean J Ophthalmol* 2011, 25(1):37-41

259. Monteiro ML, Hokazono K, Cunha LP, Oyamada MK: Multifocal pattern electroretinography for the detection of neural loss in eyes with permanent temporal hemianopia or quadrantanopia from chiasmal compression. *British Journal of Ophthalmology* 2012, 96(1):104-9

260. Ohkubo S, Higashide T, Takeda H, Murotani E, Hayashi Y, Sugiyama K: Relationship between macular ganglion cell complex parameters and visual field parameters after tumor resection in chiasmal compression. *Jpn J Ophthalmol* 2012, 56(1):68-75

261. Monteiro ML, Hokazono K, Cunha LP, Oyamada MK: Correlation between multifocal pattern electroretinography and Fourier-domain OCT in eyes with temporal hemianopia from chiasmal compression. *Graefes Arch Clin Exp Ophthalmol* 2013, 251(3):903-15

262. Monteiro ML, Afonso CL: Macular thickness measurements with frequency domain-OCT for quantification of axonal loss in chronic papilledema from pseudotumor cerebri syndrome. *Eye* 2014, 28(4):390-8

263. Monteiro ML, Hokazono K, Fernandes DB, Costa-Cunha LV, Sousa RM, Raza AS, Wang DL, Hood DC: Evaluation of inner retinal layers in eyes with temporal hemianopic visual loss from chiasmal compression using optical coherence tomography. *Invest Ophthalmol Vis Sci* 2014, 55(5):3328-36

264. Fonseca PL, Rigamonti D, Miller NR, Subramanian PS: Visual outcomes of surgical intervention for pseudotumour cerebri: optic nerve sheath fenestration versus cerebrospinal fluid diversion. *British Journal of Ophthalmology* 2014, 98(10):1360-3

265. Afonso CL, Raza AS, Kreuz AC, Hokazono K, Cunha LP, Oyamada MK, Monteiro ML: Relationship between pattern electroretinogram, frequency-domain OCT, and automated perimetry in chronic papilledema from pseudotumor cerebri syndrome. *Invest Ophthalmol Vis Sci* 2015, 56(6):3656-65

266. Afonso CL, Talans A, Monteiro MLR: Factors affecting visual loss and visual recovery in patients with pseudotumor cerebri syndrome. *Arq Bras Oftalmol* 2015, 78(3):175-9

267. Sousa RM, Oyamada MK, Cunha LP, Monteiro MLR: Multifocal visual evoked potential in eyes with temporal hemianopia from chiasmal compression: correlation with standard autormated perimetry and OCT findings. *Invest Ophthalmol Vis Sci* 2017, 58(11):4436-49

268. Jones J, Ruge J: Intraoperative magnetic resonance imaging in pituitary macroadenoma surgery: an assessment of visual outcome. *Neurosurg* 2007, 23(5) DOI: 10.3171/FOC-07/11/E12.

269. Cannavo S, De Natale R, Curto L, Li Calzi L, Trimarchi F: Effectiveness of computer-assisted perimetry in the follow-up of patients with pituitary microadenoma responsive to medical treatment. *Clin Endocrinol (Oxf)* 1992, 37(2):157-61

270. Grochowicki M, Khalfallah Y, Vighetto A, Berquet S, Sassolas G: Ophthalmic results in patients with macroprolactinomas treated with a new prolactin inhibitor CV 205-502. *British Journal of Ophthalmology* 1993, 77(12):785-8

271. Kupersmith MJ, Rosenberg C, Kleinberg D: Visual loss in pregnant women with pituitary adenomas. *Ann Intern Med* 1994, 121(7):473-7

272. Ikeda H, Yoshimoto T: Visual disturbances in patients with pituitary adenoma. *Acta Neurologica Scandinavica* 1995, 92(2):157-60

273. Kaur A, Banerji D, Kumar D, Sharma K: Visual status in suprasellar pituitary tumours. *Indian Journal of Ophthalmology* 1995, 43(3):131-4

274. Burgett RA, Purvin VA, Kawasaki A: Lumboperitoneal shunting for pseudotumor cerebri. *Neurology* 1997, 49(3):734-9

275. Colao A, Cerbone G, Cappabianca P, Ferone D, Alfieri A, Di Salle F, Faggiano A, Merola B, de Divitiis E, Lombardi G: Effect of surgery and radiotherapy on visual and endocrine function in nonfunctioning pituitary adenomas. *J Endocrinol Invest* 1998, 21(5):284-90

276. Colao A, Di Sarno A, Landi ML, Cirillo S, Sarnacchiaro F, Facciolli G, Pivonello R, Cataldi M, Merola B, Annunziato L *et al*: Long-term and low-dose treatment with cabergoline induces macroprolactinoma shrinkage. *J Clin Endocrinol Metab* 1997, 82(11):3574-9

277. Huang WC, Lee LS: Visual field defects in patients with pituitary adenomas. *Chung Hua I Hsueh Tsa Chih* 1997, 60(5):245-51

278. Nobels FR, de Herder WW, van den Brink WM, Kwekkeboom DJ, Hofland LJ, Zuyderwijk J, de Jong FH, Lamberts SW: Long-term treatment with the dopamine agonist quinagolide of patients with clinically non-functioning pituitary adenoma. *Eur* 2000, 143(5):615-21

279. Rivoal O, Brezin AP, Feldman-Billard S, Luton JP: Goldmann perimetry in acromegaly: a survey of 307 cases from 1951 through 1996. *Ophthalmology* 2000, 107(5):991-7

280. Asano S, Ueki K, Suzuki I, Kirino T: Clinical features and medical treatment of male prolactinomas. *Acta Neurochir (Wien)* 2001, 143(5):465-70

281. Colao A, Sarno AD, Cappabianca P, Briganti F, Pivonello R, Somma CD, Faggiano A, Biondi B, Lombardi G: Gender differences in the prevalence, clinical features and response to cabergoline in hyperprolactinemia. *Eur* 2003, 148(3):325-31

282. Corsello SM, Ubertini G, Altomare M, Lovicu RM, Migneco MG, Rota CA, Colosimo C: Giant prolactinomas in men: efficacy of cabergoline treatment. *Clin Endocrinol (Oxf)* 2003, 58(5):662-70

283. Chattopadhyay A, Bhansali A, Masoodi SR: Long-term efficacy of bromocriptine in macroprolactinomas and giant prolactinomas in men. *Pituitary* 2005, 8(2):147-54

284. Carrim ZI, Reeks GA, Chohan AW, Dunn LT, Hadley DM: Predicting impairment of central vision from dimensions of the optic chiasm in patients with pituitary adenoma. *Acta Neurochir (Wien)* 2007, 149(3):255-60

285. Kitthaweesin K, Ployprasith C: Ocular manifestations of suprasellar tumors. *J Med Assoc Thai* 2008, 91(5):711-5

286. Wang H, Sun W, Fu Z, Si Z, Zhu Y, Zhai G, Zhao G, Xu S, Pang Q: The pattern of visual impairment in patients with pituitary adenoma. *J Int Med Res* 2008, 36(5):1064-9

287. Imran SA, Fleetwood IG, O'Connell CM, Ransom TP, Mulroy LA, Ur E, Clarke DB: Outcome of stereotactic radiotherapy for patients with uncontrolled acromegaly. *Can J Neurol Sci* 2009, 36(4):468-74

288. Shen MQ, Ye W, Zhang YY, Chen J: [Visual field defects in 169 cases of pituitary adenomas]. *Chung-Hua Yen Ko Tsa Chih* 2009, 45(12):1074-9

289. Aui-aree N, Phruanchroen C, Oearsakul T, Hirunpat S, Sangthong R: Three years experience of suprasellar tumors in neuro-ophthalmology clinic. *J Med Assoc Thai* 2010, 93(7):818-23

290. Berkmann S, Fandino J, Zosso S, Killer HE, Remonda L, Landolt H: Intraoperative magnetic resonance imaging and early prognosis for vision after transsphenoidal surgery for sellar lesions. *Journal of Neurosurgery* 2011, 115(3):518-27 DOI: 10.3171/2011.4.JNS101568.

291. Lee MJ, Hwang JM: Initial visual field as a predictor of recurrence and postoperative visual outcome in children with craniopharyngioma. *J Pediatr Ophthalmol Strabismus* 2012, 49(1):38-42

292. Schmalisch K, Milian M, Schimitzek T, Lagreze WA, Honegger J: Predictors for visual dysfunction in nonfunctioning pituitary adenomas - implications for neurosurgical management. *Clin Endocrinol (Oxf)* 2012, 77(5):728-34

293. Lee S, Kim SJ, Yu YS, Kim YH, Paek SH, Kim DG, Jung HW: Prognostic factors for visual recovery after transsphenoidal pituitary adenectomy. *Br J Neurosurg* 2013, 27(4):425-9

294. Astradsson A, Munck Af Rosenschold P, Feldt-Rasmussen U, Poulsgaard L, Wiencke AK, Ohlhues L, Engelholm SA, Broholm H, Hansen Moller E, Klose M *et al*: Visual outcome, endocrine function and tumor control after fractionated stereotactic radiation therapy of craniopharyngiomas in adults: findings in a prospective cohort. *Acta Oncol* 2017, 56(3):415-21

295. Astradsson A, Wiencke AK, Munck af Rosenschold P, Engelholm SA, Ohlhues L, Roed H, Juhler M: Visual outcome after fractionated stereotactic radiation therapy of benign anterior skull base tumors. *J Neurooncol* 2014, 118(1):101-8

296. Degerliyurt A, Teber S, Karakaya G, Guven A, Seker ED, Arhan EP, Sayli TR: Pseudotumor cerebri/idiopathic intracranial hypertension in children: an experience of a tertiary care hospital. *Brain Dev* 2014, 36(8):690-9

297. Drimtzias E, Falzon K, Picton S, Jeeva I, Guy D, Nelson O, Simmons I: The ophthalmic natural history of paediatric craniopharyngioma: a long-term review. *J Neurooncol* 2014, 120(3):651-6

298. Yoneoka Y, Hatase T, Watanabe N, Jinguji S, Okada M, Takagi M, Fujii Y: Early morphological recovery of the optic chiasm is associated with excellent visual outcome in patients with compressive chiasmal syndrome caused by pituitary tumors. *Neurol Res* 2015, 37(1):1-8

299. Shimon I, Sosa E, Mendoza V, Greenman Y, Tirosh A, Espinosa E, Popovic V, Glezer A, Bronstein MD, Mercado M: Giant prolactinomas larger than 60 mm in size: a cohort of massive and aggressive prolactin-secreting pituitary adenomas. *Pituitary* 2016, 19(4):429-36

300. Hudson H, Rissell C, Gauderman WJ, Feldon SE: Pituitary tumor volume as a predictor of postoperative visual field recovery. Quantitative analysis using automated static perimetry and computed tomography morphometry. *J Clin Neuroophthalmol* 1991, 11(4):280-3

301. Tang Y, Qu YZ, Yang L, Wang J, Wang LN, Fang M, Lu W: [Assessing the damage to visual function by optical coherence tomography and the visual field test in Saddle area tumor patients]. *Chung-Hua Yen Ko Tsa Chih* 2012, 48(11):1001-4

302. Poon A, McNeill P, Harper A, O'Day J: Patterns of visual loss associated with pituitary macroadenomas. *Australian & New Zealand Journal of Ophthalmology* 1995, 23(2):107-15

303. Frisen L, Jensen C: How robust is the optic chiasm? Perimetric and neuro-imaging correlations. *Acta Neurologica Scandinavica* 2008, 117(3):198-204

304. Abouaf L, Vighetto A, Lebas M: Neuro-ophthalmologic exploration in non-functioning pituitary adenoma. *Ann Endocrinol (Paris)* 2015, 76(3):210-9

305. Garcia T, Sanchez S, Litre CF, Radoi C, Delemer B, Rousseaux P, Ducasse A, Arndt C: Prognostic value of retinal nerve fiber layer thickness for postoperative peripheral visual field recovery in optic chiasm compression. *Journal of Neurosurgery* 2014, 121(1):165-9

306. Jacob M, Raverot G, Jouanneau E, Borson-Chazot F, Perrin G, Rabilloud M, Tilikete C, Bernard M, Vighetto A: Predicting visual outcome after treatment of pituitary adenomas with optical coherence tomography. *American Journal of Ophthalmology* 2009, 147(1):64-70

307. Schiefer U, Isbert M, Mikolaschek E, Mildenberger I, Krapp E, Schiller J, Thanos S, Hart W: Distribution of scotoma pattern related to chiasmal lesions with special reference to anterior junction syndrome. *Graefes Arch Clin Exp Ophthalmol* 2004, 242(6):468-77

308. Unsold R: [Ophthalmological symptoms of idiopathic intracranial hypertension: Importance for diagnosis and clinical course]. *Ophthalmologe* 2015, 112(10):808-13

309. Mohr G, Hardy J, Comtois R, Beauregard H: Surgical management of giant pituitary adenomas. *Can J Neurol Sci* 1990, 17(1):62-6

310. Comtois R, Beauregard H, Somma M, Serri O, Aris-Jilwan N, Hardy J: The clinical and endocrine outcome to trans-sphenoidal microsurgery of nonsecreting pituitary adenomas. *Cancer* 1991, 68(4):860-6

311. Cury ML, Fernandes JC, Machado HR, Elias LL, Moreira AC, Castro M: Non-functioning pituitary adenomas: clinical feature, laboratorial and imaging assessment, therapeutic management and outcome. *Arq Bras Endocrinol Metabol* 2009, 53(1):31-9

312. Campbell PG, McGettigan B, Luginbuhl A, Yadla S, Rosen M, Evans JJ: Endocrinological and ophthalmological consequences of an initial endonasal endoscopic approach for resection of craniopharyngiomas. *Neurosurg* 2010, 28(4):E8 DOI: 10.3171/2010.1.FOCUS09292.

313. Foroozan R: Visual Findings in Chiasmal Syndromes. *International Ophthalmology Clinics* 2015, 56(1):1-27

314. Bandyopadhyay S, Jacobson DM: Clinical features of late-onset pseudotumor cerebri fulfilling the modified dandy criteria. *Journal of Neuro-Ophthalmology* 2002, 22(1):9-11

315. Gilhotra JS, Mitchell P, Healey PR, Cumming RG, Currie J: Homonymous visual field defects and stroke in an older population. *Stroke* 2002, 33(10):2417-20

316. Bouwmeester L, Heutink J, Lucas C: The effect of visual training for patients with visual field defects due to brain damage: a systematic review. *Journal of Neurology, Neurosurgery and Psychiatry* 2007, 78(6):555-64

317. Kedar S, Zhang X, Lynn MJ, Newman NJ, Biousse V: Pediatric Homonymous Hemianopia. *Journal of American Association for Pediatric Ophthalmology and Strabismus* 2006, 10(3):249-52

318. Townend BS, Sturm JW, Petsoglou C, O'Leary B, Whyte S, Crimmins D: Perimetric homonymous visual field loss post-stroke. *Journal of Clinical Neuroscience* 2007, 14(8):754-6

319. Zhang X, Kedar S, Lynn MJ, Newman NJ, Biousse V: Homonymous hemianopias: Clinical-anatomic correlations in 904 cases. *Neurology* 2006, 66(6):906-10

320. Kedar S, Zhang X, Lynn MJ, Newman NJ, Biousse V: Congruency in homonymous hemianopia. *American Journal of Ophthalmology* 2007, 143(5):772-80

321. Taravati P, Woodward KR, Keltner JL, Johnson CA, Redline D, Carolan J, Huang CQ, Wall M: Sensitivity and specificity of the Humphrey Matrix to detect homonymous hemianopias. *Invest Ophthalmol Vis Sci* 2008, 49(3):924-8

322. Ogawa K, Ishikawa H, Suzuki Y, Oishi M, Kamei S: Clinical study of the visual field defects caused by occipital lobe lesions. *Cerebrovascular Diseases* 2014, 37:102-8

323. Cavanaugh MR, Huxlin KR: Visual discrimination training improves Humphrey perimetry in chronic cortically induced blindness. *Neurology* 2017, 88(19):1856-64

324. Celebisoy M, Celebisoy N, Bayam E, Köse T, Çelebisoy M, Çelebisoy N, Bayam E, Köse T: Recovery of visual-field defects after occipital lobe infarction: a perimetric study. *Journal of Neurology, Neurosurgery & Psychiatry* 2011, 82(6):695-702 DOI: 10.1136/jnnp.2010.214387.

325. Rowe FJ, Wright D, Brand D, Jackson C, Harrison S, Maan T, Scott C, Vogwell L, Peel S, Akerman N *et al*: A prospective profile of visual field loss following stroke: prevalence, type, rehabilitation and outcome. *BioMed Research International* 2013, 2013 DOI: 10.1155/2013/719096.

326. Koenraads Y, Porro GL, Braun KP, Groenendaal F, de Vries LS, van der Aa NE: Prediction of visual field defects in newborn infants with perinatal arterial ischemic stroke using early MRI and DTI-based tractography of the optic radiation. *Europ J Paediatr Neurol* 2016, 20(2):309-18

327. Isa K, Miyashita K, Yanagimoto S, Nagatsuka K, Naritomi H: Homonymous Defect of Macular Vision in Ischemic Stroke. *European Neurology* 2001, 46(3):126-30

328. Lepore FE: The preserved temporal crescent: The clinical implications of an “endangered” finding. *Neurology* 2001, 57(10):1918-21

329. Bergsma DP, van der Wildt G: Visual training of cerebral blindness patients gradually enlarges the visual field. *British Journal of Ophthalmology* 2010, 94(1):88-96

330. Falke P, Abela Jr. BM, Krakau CE, Lilja B, Lindgarde F, Maly P, Stavenow L: High frequency of asymptomatic visual field defects in subjects with transient ischaemic attacks or minor strokes. *J Intern Med* 1991, 229(6):521-5

331. Fedorov A, Jobke S, Bersnev V, Chibisova A, Chibisova Y, Gall C, Sabel BA: Restoration of vision after optic nerve lesions with noninvasive transorbital alternating current stimulation: a clinical observational study. *Brain Stimul* 2011, 4(4):189-201

332. Benshir MD: Prevalence of vision disorders after stroke: a pilot study to identify the visual needs of stroke patients. *Optometry & Visual Performance* 2016, 4(4):128-32

333. Cassidy TP, Bruce DW, Gray CS: Visual field loss after stroke: confrontation and perimetry in the assessment of recovery. *Journal of Stroke and Cerebrovascular Diseases* 2001, 10(3):113-7

334. Suchoff IB, Kapoor N, Ciuffreda KJ, Rutner D, Han E, Craig S: The frequency of occurrence, types, and characteristics of visual field defects in acquired brain injury: a retrospective analysis. *Optometry* 2008, 79(5):259-65

335. Mills RP: Automated perimetry in neuro-ophthalmology. *International Ophthalmology Clinics* 1991, 31(4):51-70

336. Keltner JL, Johnson CA: Short-wavelength automated perimetry in neuro-ophthalmologic disorders. *Archives of Ophthalmology* 1995, 113(4):475-81

337. Donahue SP: Perimetry techniques in neuro-ophthalmology. *Curr Opin Ophthalmol* 1999, 10(6):420-8

338. Munoz Negrete FJ, Rebolleda G: Automated perimetry and neuro-ophthalmology. Topographic correlation. *Arch Soc Esp Oftalmol* 2002, 77(8):413-28

339. Zhang X, Kedar S, Lynn MJ, Newman NJ, Biousse V: Homonymous hemianopia in stroke. *Journal of Neuro-Ophthalmology* 2006, 26(3):180-3

340. Yoon MK, Hwang TN, Day S, Hong J, Porco T, McCulley TJ: Comparison of Humphrey Matrix frequency doubling technology to standard automated perimetry in neuro-ophthalmic disease. *Middle East Afr J Ophthalmol* 2012, 19(2):211-5

341. McCoy AN, Quigley HA, Wang J, Miller NR, Subramanian PS, Ramulu PY, Boland MV: Development and validation of an improved neurological hemifield test to identify chiasmal and postchiasmal lesions by automated perimetry. *Invest Ophthalmol Vis Sci* 2014, 55(2):1017-23

342. Boland MV, McCoy AN, Quigley HA, Miller NR, Subramanian PS, Ramulu PY, Murakami P, Danesh-Meyer HV: Evaluation of an algorithm for detecting visual field defects due to chiasmal and postchiasmal lesions: the neurological hemifield test. *Invest Ophthalmol Vis Sci* 2011, 52(11):7959-65
